# Synthetic cannabinoids in e‐cigarettes seized from English schools

**DOI:** 10.1111/add.70110

**Published:** 2025-06-26

**Authors:** Gyles E. Cozier, Matthew Gardner, Sam Craft, Martine Skumlien, Jack Spicer, Rachael Andrews, Alexander Power, Tom Haines, Richard Bowman, Amy E. Manley, Peter Sunderland, Oliver B. Sutcliffe, Stephen M. Husbands, Lindsey Hines, Gillian Taylor, Tom P. Freeman, Jennifer Scott, Christopher R. Pudney

**Affiliations:** ^1^ Department of Life Sciences University of Bath Bath UK; ^2^ Department of Psychology University of Bath Bath UK; ^3^ Department of Social and Policy Science University of Bath Bath UK; ^4^ Department of Computer Science University of Bath Bath UK; ^5^ School of Physics and Astronomy University of Glasgow Glasgow UK; ^6^ Bristol Medical School University of Bristol Bristol UK; ^7^ MANchester DRug Analysis and Knowledge Exchange (MANDRAKE), Department of Natural Sciences Manchester Metropolitan University Manchester UK; ^8^ School of Health and Life Sciences Teesside University Middlesbrough UK; ^9^ Centre for Academic Primary care, Bristol Medical School University of Bristol Bristol UK; ^10^ Centre for Therapeutic Innovation University of Bath Bath UK

**Keywords:** e‐cigarette, K2, school, spice, synthetic cannabinoids, THC, vaping

## Abstract

**Background and aims:**

People who use synthetic cannabinoids (SCs) report debilitating side effects and withdrawal symptoms, coupled with dependence. In the UK, SC use was believed to be largely restricted to prison, where they are the most common drug and associated with nearly half of non‐natural deaths, or poly‐drug users in the community who are also likely to be homeless. However, national media reporting has increasingly identified cases of children collapsing in schools, which are claimed to be associated with vaping and putatively involving a drug such as delta‐9‐tetrahydrocannabinol (THC) or SCs. We therefore conducted the first study to identify and quantify SCs in e‐cigarettes routinely collected from schools in England.

**Design:**

E‐cigarette and e‐liquid samples seized by teachers in schools were identified through engagement with police forces and city councils in England. We sought agreements across broad geographical areas and based on acquiring the relevant approvals at a local level. Sample bias is considered in the analysis and reporting.

**Setting and cases:**

Samples were submitted from 27 secondary (age 11–18) schools from geographically distinct regions of England, representing a broad range of social metrics (free school meals, persistent absenteeism and special educational needs). All submitted samples were anonymised and no identifying information was collected. Analysis of samples was conducted both in a laboratory setting and in‐field at local police stations.

**Measurements:**

Qualitative gas chromatography–mass spectrometry and liquid chromatography–mass spectrometry were used to identify SCs and THC in e‐cigarettes/liquid, with concentration measured by quantitative nuclear magnetic resonance spectroscopy. A subset of samples was screened for SCs and THC using a portable detector based on combined fluorescence and photochemical discrimination.

**Findings:**

E‐cigarettes containing SC were identified in 77.8% of all participating schools and were detected in 17.4% of all samples seized. These were almost entirely in refillable devices and liquid bottles, with very few in single use products. The percentage of SC e‐cigarettes in schools positively correlated with the fraction of pupils eligible for free school meals, a social deprivation metric (Pearson's correlation r = 0.65 and *P* = 0.003). Positive samples contained a median SC concentration of 0.42 (interquartile range = 0.77) mg mL^−1^ with a maximum of 3.6 mg mL^−1^. In contrast, few samples contained THC (1.2%).

**Conclusions:**

E‐cigarettes containing synthetic cannabinoids were identified in three quarters of 27 secondary schools in England that were sampled.

## INTRODUCTION

Synthetic cannabinoids (SCs), often referred to as spice or K2, are a large class of synthetic drugs whose structure rapidly changes [1, 2]. SCs are highly potent, often acting as full cannabinoid receptor agonists [3]. However, their structure is dissimilar to typical cannabinoids found in cannabis, such as Δ‐9‐tetrahydrocannabinol (THC), which acts as a partial cannabinoid receptor agonist [4] and has a considerably lower risk profile compared to SCs [5]. Potential consequences of SC use include psychosis, seizures, hypertensive crisis and death [6]. There is only sparse research into the correlation between different SC structures and their pharmacology and risk profile [7]. Indeed, there are now a number of studies that point to SCs potentially having effects at sites other than cannabinoid receptors [3, 8, 9].

In the United Kingdom, data suggest that use of SCs [classed as a novel psychoactive substances (NPS)] in the general population is extremely low, with only 0.5% of adults (age 16–59) in England and Wales reported using a NPS in the year to March 2024 [10]. However, SCs are the dominant drug used in the British prison system [11, 12] and are commonly used by people who are homeless [13]. These are considered at‐risk individuals with complex poly‐substance use histories. People who use SCs report highly variable and unpredictable effects, which increase the risk of collapsing and becoming comatose [14]. Nearly half of all non‐natural deaths in British prisons have been associated with SC use [14]. Users report strong withdrawal symptoms on discontinuing use, which are more severe than for cannabis [13, 15, 16].

The use of e‐cigarettes (both nicotine and nicotine free) has become common in England. There have been growing reports of SCs being found in e‐cigarette liquid. Between April 2023 and March 2024, testing by the UK drug checking service, Welsh Emerging Drugs and Identification of Novel Substances (WEDINOS), showed that 41% of 211 submitted e‐cigarettes contained SCs [17]. Crucially, none of these samples were submitted with the purchase intent of SCs.

SC e‐cigarette liquid is inexpensive, a snapshot survey ending in January 2023 showed SCs being sold for as little as £1.60 mL^−1^ (€1.86/$1.98—January 2023 exchange rates) and available on‐line [18]. The reported concentration of the SC e‐cigarettes varies from approximately 1 mg mL^−1^ up to a maximum reported value of 24.1 mg mL^−1^ [19], although there have been relatively few studies, so the range maybe be greater.

**FIGURE 1:**
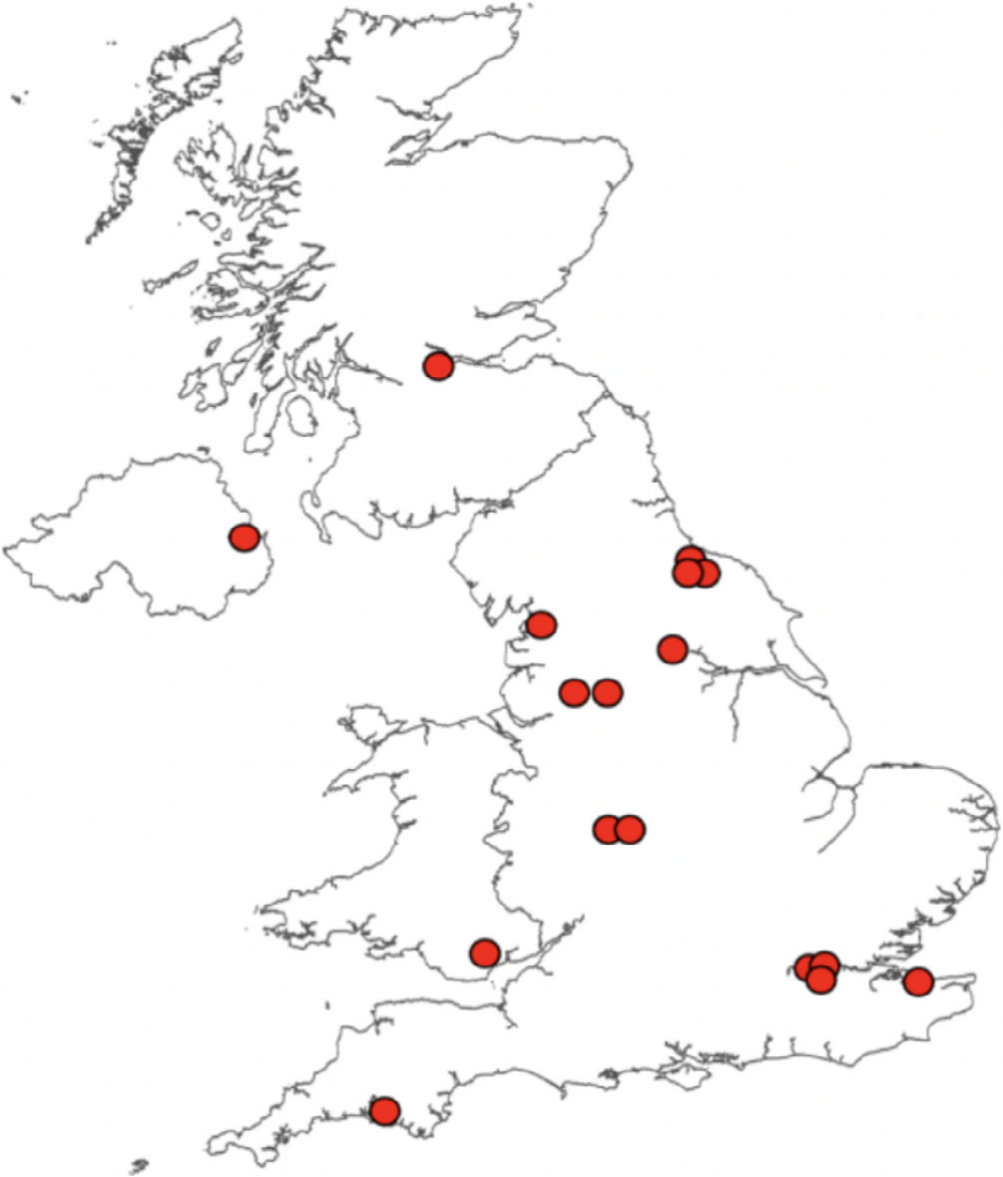
Media reports of adverse effects in schools associated with vaping and putatively associated with synthetic cannabinoids (SC) use.

The use of e‐cigarettes has extended to young people with 9% of 11 to 15 year‐olds (19% for just 15 year‐olds) reporting using e‐cigarettes in 2023 [20]. In comparison, 3% of 11 to 15 year‐olds (7% for just 15 year‐olds) currently smoke [20]. In the last year, media reported over 16 putative SC/THC related adverse effects associated with vaping in British schools (Figure 1 and Table S1), and we are aware of many more from personal communications from police forces across the United Kingdom. These unconfirmed reports highlight there is a fundamental lack of analytically confirmed data to assess the actual occurrence of SC e‐cigarettes in schools.

THC overdose requires consumption of very high quantities, suggesting that the reported adverse effects are caused by something else. The risk of emergency medical treatment is estimated to be 30‐fold greater for use of SCs compared to THC [5]. Therefore, we hypothesise that school children maybe using e‐cigarettes containing SCs, and this could be associated with some of the adverse effects reported. Here, we sampled seized e‐cigarettes from secondary (age 11–18) schools across a range of regions in England, assaying for the presence and concentration of substances controlled under the Misuse of Drugs Act (1971) [21] and the Psychoactive Substances Act (2016) [22]. Further, we demonstrate how a recent innovation in portable SC detection can be effectively used to monitor the presence of SC e‐cigarettes at a local level.

## METHODS

### Region selection

Our rationale for collecting samples was the occurrence of a media report of SC intoxication. The local police forces that had jurisdiction for those regions were contacted to seek permission to sample, and local councils were consulted to allow the sampling to proceed. Permissions were obtained for three regions, R1–R3, whereas for other requests either the police forces did not wish to participate during the time period of the study, or the local council was not willing to allow the sampling to proceed. R1 and R2 (13 and 9 schools, respectively) are single regions, while R3 comprises of five schools drawn from two separate metropolitan regions (1 and 4 schools in each region). We include a final region R4 (4 schools), but we had a different agreement with the police as described below.

Because of constraints and limitations from the local police forces and local councils we could not adopt a consistent methodology of collection or analysis across all regions (details described below). We have anonymised the specific regions sampled at the request of the police forces involved and to protect the anonymity of the schools, but R1–R4 represent a very large North–South length span of England (250 miles), consisting of five different regions in the United Kingdom, all of which are at least 90 miles apart. We note that the analysis was not pre‐registered and that the results should be considered exploratory.

### Sample acquisition

For R1–R3, the regional police force requested local schools to submit seized e‐cigarettes/e‐liquids and participation was voluntary. We stress that the submissions were unbiased, no specific schools were targeted, no specific selection protocol for identifying samples was defined, and the seized material was identified by teachers working in each school based on their usual procedures. The seizures were during September 2023 to June 2024, effectively a school year. The police force collated all submitted samples without a further selection step.

For R1–R2, the relevant police force conveyed the samples to our laboratories for analysis. For R3, we collected data from samples at a local police station in R3 using our portable technology described below. Any positives were immediately handed to a police officer who continuously chaperoned the testing.

Sampling for R4 was conducted over the period November 2023 to July 2024 where the samples were considered biased as they either were considered suspicious and schools/police forces requested analysis, or a subjective/objective triage step had been applied. Therefore, these data are a separate validation of the general findings from R1–R3 to establish whether the findings are borne out in a random region. Samples were conveyed to our laboratories for analysis by the police.

### Sample analysis

Details of analytical methods are described in the Supporting information. Samples from R1 and R2 were analysed by liquid chromatography–mass spectrometry (LC–MS) to identify if they contained any substance controlled under the Misuse of Drugs Act (1971) [21] and the Psychoactive Substances Act (2016) [22] by searches against HighResNPS and ForTox mass spectral databases. Figures S1 and S2 show representative LC–MS data for positive samples, with the identifying parent molecules/fragments ions listed in Table S2. Structures of controlled substances identified are shown in Figure S3. Where possible, quantitative nuclear magnetic resonance spectroscopy (qNMR) was used to quantify the drug concentration. Figures S4–S7 show example NMR spectra and Figure S8 shows the standard concentration plot. The accurate detection limit of our qNMR methodology is approximately 50 μg mL^−1^, and samples below this level are described as ‘low’. R3 samples were retained by the police after testing using our portable technology, therefore, no further analysis was possible. Samples from R4 were analysed by gas chromatography–mass spectrometry (GC–MS) by comparison to standards, NIST database and Cayman Chemicals forensic database. All samples were visually analysed to record data about brand type, flavour, liquid colour and form factor [single use (SU), refillable (RF), labelled bottle (LB) and unlabelled bottle (UB)]. We define a SU as one where it is not designed to be refilled.

### Portable sampling method

We have reported a portable device for the generic detection of SCs and THC from sealed e‐cigarettes and e‐cigarette liquids [23, 24]. The technology self‐actuates an e‐cigarette and extracts a small amount of vapour, condensed on a solid matrix with subsequent detection based on combined fluorescence and photochemical discrimination. For e‐liquids a few drops can be placed on the solid matrix for discrimination. We reasoned this tool would be useful to expand data sampling.

Using sample sets from R1 and R2 for calibration and previous work with THC [24], we tuned the device to have a limit of detection (LOD) of 0.3 mg mL^−1^ for SCs and 5 mg mL^−1^ for THC. This gave an accuracy of 95% for R1 and R2 samples (statistics given in Table 1). The device accuracy will decrease below the LOD. The calibrated device was used to sample the seized e‐cigarettes/e‐liquids from R3 to identify those that contained a SC or THC.

**TABLE 1:** Usage statistics of the presumptive device.

Raw data	
True positive	48
True negative	42
False positive	2
False negative	3
Total	95

Resulting statistics (%)	
Sensitivity	94
Specificity	95
Accuracy	95

### School demographics

The Government of the United Kingdom (Gov.UK) publishes performance details of individual schools in England [25] and includes the following characteristics of the schools that can be used to examine if there is any correlation with the occurrence of SC containing e‐cigarettes:

- Persistent absence—percentage of pupils missing 10% or more of the mornings or afternoons they could attend.
- Pupils with special educational needs (SEN) support.
- Pupils with a SEN education, health and care plan (EHCP).
- Pupils eligible for free school meals at any time during the past 6 years (FSM).

Data for these characteristics for the 2023 to 2024 school year were averaged for the schools in each region R1–R3 and compared to all state funded secondary schools in England (Table 2). As described above, sampling in R4 was biased, therefore, we did not include those schools in this part of the study. Persistent absence data for 2023 to 2024 were not released at the time of study, therefore, 2022 to 2023 data were used. A postcode‐based metric of social deprivation was not used as schools tend to draw widely across an area, and therefore, we did not feel this would be useful or representative. Instead we use the FSM for this purpose.

**TABLE 2:** School demographics in the selected regions.

	England^a^	R1	R2	R3	R1 + R2 + R3
No. of schools	3452	13	9	5	27 total
FSM	25.7 (19.9)%	30.5 (16.7)%	28.2 (32.4)%	31.1 (13.3)%	29.4 (22.7)%
Persistent absence	26.5%	25.7 (14.5)%	28.3 (13.0)%	27.9 (9.2)%	26.5 (10.6)%
SEN support	13.2 (6.7)%	12.9 (10.2)%	12.7 (7.2)%	14.4 (4.2)%	12.8 (7.3)%
EHCP	2.6 (2.0)%	2.9 (0.8)%	2.6 (0.7)%	3.2 (0.9)%	2.7 (1.1)%

*Note*: Data obtained from Gov.UK [25].

Abbreviations: EHCP, education, health and care plan; FSM, free school meals; IQR, interquartile range; SEN, special educational needs; UK, United Kingdom.

^a^ England state funded secondary schools. Percentages are median averages with (IQR). Full data for persistent absence was not available, therefore, the UK government quoted average was used (no IQR published).

### Statistical analysis

Descriptive statistics (conducted in Excel) were used to summarise controlled drug occurrence in the different regions, how the SC positives (both total numbers and concentration) related to form factor (SU, RF, LB, UB) and e‐liquid colour and to compare school characteristics in R1–R3 with England averages. Data for school characteristics and from qNMR were non‐normally distributed, therefore, median concentration and interquartile range (IQR) were used. Data for persistent absence was not available separately for England secondary schools, therefore, the UK government quoted average is used (no IQR reported). The ‘low’ concentration samples from qNMR were excluded from median/IQR calculations when comparing concentrations of single form factors, because of insufficient sample numbers.

Linear regression and Pearson's correlation analysis were used to look for correlation between school characteristics and presence of SC e‐cigarettes. In an effort to decrease sampling bias we only included schools where there are >20 submitted samples (4 schools from R1, 5 schools from R2 and 2 from R3). The data represents 82% of all the seized e‐cigarettes reported in this study. The analysis was performed in OriginPro and Excel.

## RESULTS

### E‐cigarette/e‐liquid samples collected

In total, 512 samples were collected from R1–R3 (R1 = 156, R2 = 272, R3 = 85) (Table 3). These data represent, as close as possible, an unbiased sampling of e‐cigarettes from 27 schools. Tables S3–S5 show the data for all samples broken down to individual school level. Additionally, 50 samples that were thought of as suspicious were collected from the four schools in R4 (Table S6). Table S7 summarises number of samples and positive detections for each school in R1–R3.

**TABLE 3:** The prevalence of occurrence of SC and THC in schools from selected regions.

	R1	R2	R3^a^	R1 + R2 + R3 combined
Total samples	156	272	84	512
Tested samples	119	251	84	454
Positive SC, % of total	35–22.4	40–14.7	14–16.7	89–17.4
Positive THC, % of total	1–0.6	0–0	5–6.0	6–1.2
SC positive	Positive (no. of form factor)			
Single use	1 (106)	7 (199)	0 (37)	8 (342)
Liquid/refill	34 (50)	33 (73)	14 (47)	81 (170)
Labelled bottle	3 (11)	0 (20)	0 (10)	3 (41)
Unlabelled bottle	11 (12)	16 (19)	9 (9)	36 (40)
Refillable device	20 (27)	17 (34)	5 (28)	42 (89)
THC positive	Positive (no. of form factor)			
Single use	1 (106)	0 (196)	2 (37)	3 (342)
Liquid/refill	0 (50)	0 (74)	3 (47)	3 (170)
Labelled liquid	0 (11)	0 (20)	0 (10)	0 (41)
Unlabelled liquid	0 (12)	0 (19)	0 (9)	0 (40)
Refillable device	0 (27)	0 (34)	3 (28)	3 (89)

Abbreviations: SC, synthetic cannabinoids; THC, Δ‐9‐tetrahydrocannabinol.

^a^ In field testing, data collected as described in the text.

### Analytical analysis of samples from R1 and R2

**FIGURE 2:**
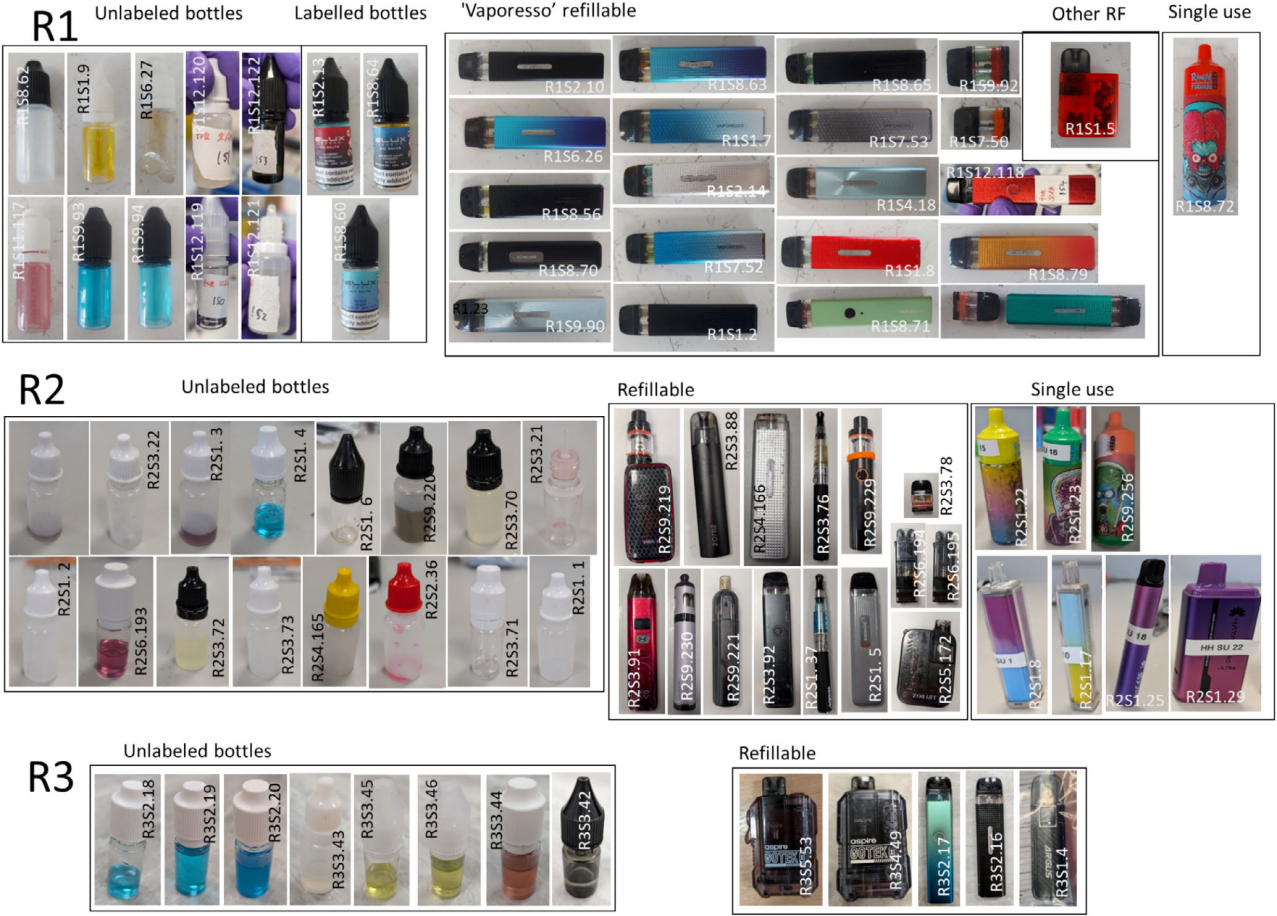
Presentation of synthetic cannabinoids (SC) e‐cigarettes from each sampling exercise (R1–R3).

E‐cigarette liquid could be extracted from 119 of 156 submitted samples from R1 and 251 of 272 from R2. Controlled drugs identified by LC–MS were six different SCs (MDMB‐4en‐PINACA, MDMB‐PINACA, ADB‐BUTINACA, MDMB‐BUTINACA, MDMB‐INACA and 4F‐MDMB‐BINACA), THC and heroin. No other SCs or controlled drugs were found. An image of all the SC and THC positive e‐cigarettes/e‐liquids are shown in Figure 2 and Figure S9, respectively.

SCs were identified in 35 (22.4%) and 40 (14.8%) samples for R1 and R2, respectively (Table 3). In R1, 1 (0.6%) THC sample was identified, with none in R2.

**FIGURE 3:**
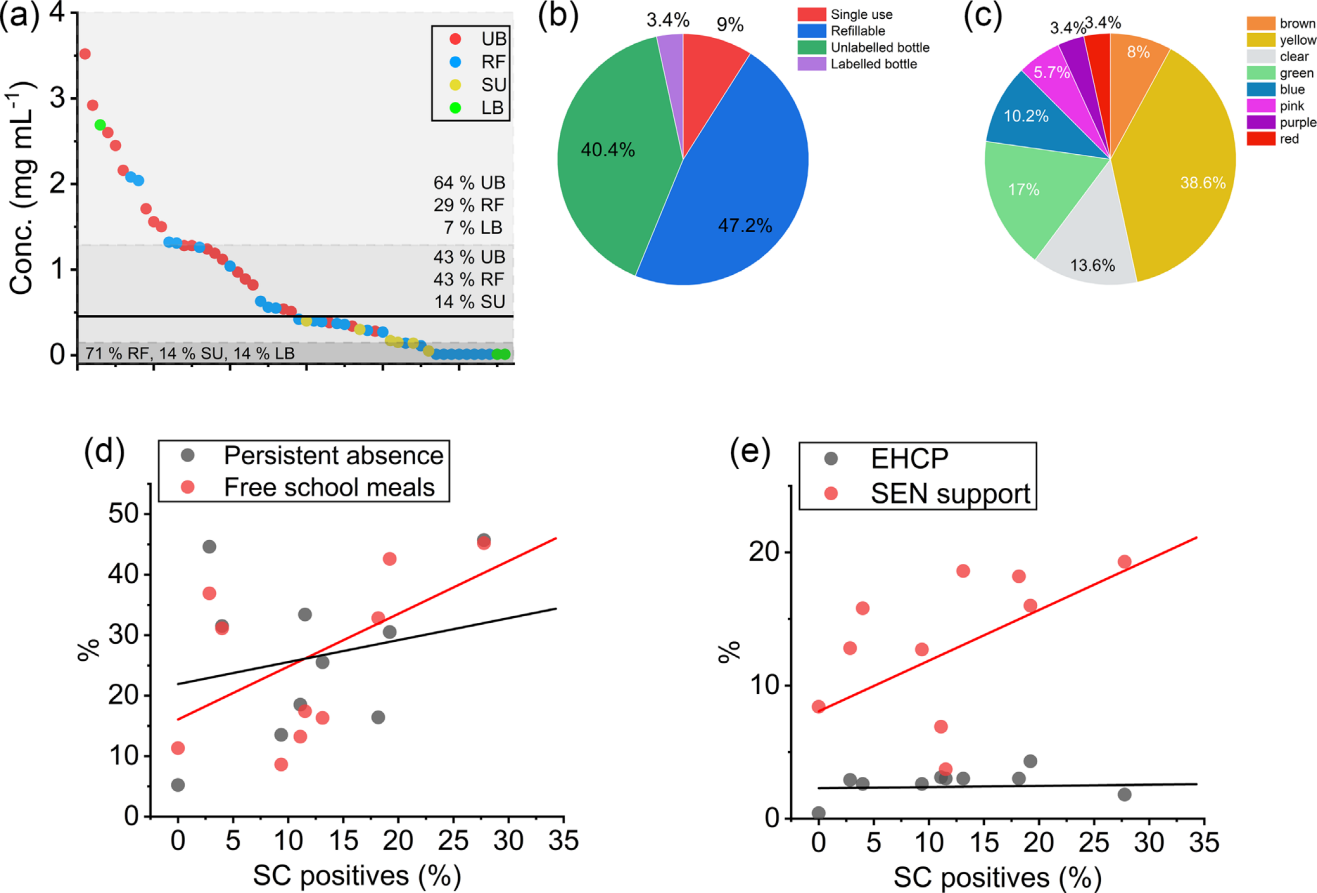
(a) Plot depicts synthetic cannabinoids (SC) concentration of each positive sample ordered from high to low and coloured by form factor. Median concentration depicted by black solid line with grey shading based on interquartile range. (b) Distribution of SC positive form factor. (c) Distribution of SC positive e‐liquid colour. (d),(e) Correlation of percentage of e‐cigarettes/e‐liquids positive for SC with school metrics for each school with >20 seized samples. Solid lines show the fit to a simple linear function and further statistical correlations are discussed in the text. SU, single use; LB, labelled bottle; UB, unlabelled bottle; RF, refillable.

In total, 76 samples contained a controlled substance, and there was sufficient material remaining in 56 for quantification by qNMR [Figure 3(c) and Tables S3 and S4]. Ten quantified samples were below the accurate detection limit of our qNMR methodology (< ~50 μg mL^−1^). These samples also did not have clear peaks observable on the LC–MS chromatograms at the dilutions used. These samples are labelled as ‘low’ in Tables S3 and S4. Sample R2S2.36 contained SCs (MDMB‐4en‐PINACA and ADB‐BUTINACA), but also a low concentration (0.1 mg mL^−1^) of heroin.

There is a broad distribution of concentrations, with a maximum of 3.6 mg mL^−1^. Combined, the samples have a median average concentration of 0.42 (IQR = 0.77) mg mL^−1^ (Table 4). The median average from R1 is 0.51 (IQR = 1.17) mg mL^−1^ and from R2 is 0.40 (IQR = 0.895) mg mL^−1^. The data cannot be fit with a single distribution function, but instead are composed of ‘low’, ‘medium’ and ‘high’ concentration distribution [Figure 3(a)]. With only 54 samples, a tri‐modal distribution cannot be accurately modelled with any degree of certainty and so Figure 3(a) shows the median and IQR distribution. The median SC concentration for UB + LB is 1.26 (IQR = 1.21) mg mL^−1^, for RF is 0.49 (IQR = 0.84) mg mL^−1^ and for SU is 0.16 (IQR = 0.13) mg ml^−1^.

**TABLE 4:** SC concentration in e‐cigarette/e‐liquid.

	R1 median (IQR) [no. of samples]	R2 median (IQR) [no. of samples]	R1 + R2 median (IQR) [no. of samples]
All samples	0.51 (1.17) [31]	0.40 (0.95) [25]	0.42 (0.77) [56]
SU	[0]	0.16 (0.13) [6]	0.16 (0.13) [6]
Liquid/refill	0.51 (1.17) [31]	0.82 (1.08) [19]	0.56 (1.03) [50]
LB + UB^a^	1.28 (0.79) [12]	1.06 (1.06) [10]	1.24 (0.89) [22]
RF^a^	0.41 (0.46) [12]	0.80 (0.77) [6]	0.49 (0.84) [18]

*Note*: Median and IQR are in mg mL^−1^.

Abbreviations: IQR, interquartile range; LB, labelled bottle; RF, refillable; SC, synthetic cannabinoids; SU, single use; UB, unlabelled bottle.

^a^ Calculated with the ‘low’ concentration samples excluded. Only a single LB (in R1, 2.69 mg mL^−1^) was above the ‘low’ concentration; there were two further ‘low’ concentration LBs in R1.

### Portable analysis of samples from R3

All 84 samples from the final unbiased region R3 were screened using the portable device. This identified 14 (16.7%) SC samples and five (6.0%) THC (Table 3 and Table S5). No SC positives were identified from single use e‐cigarettes, potentially because of the LOD of the device. Three of five of the THC e‐cigarettes were a commercial product, apparently originating in the United States, while two of five (also the THC sample identified in R1) were designed to be filled with THC oil/resin and are marketed for this purpose on available web shops.

Combining the data from R1–R3, the average percentage of all e‐cigarettes containing SCs is 17.4% (89/512). The percentage of SC e‐cigarettes is similar between R1–R3 being, 22.4, 14.8 and 16.7%, respectively. For THC the average percentage for the combined R1–R3 regions was 1.2% (6/512), which showed greater variation between regions ranging from 0% in R2 and 6.0% in R3. Of the 27 R1–R3 schools 21 (77.8%) had a positive SC e‐cigarette.

### E‐cigarette/e‐liquid characteristics analysis in R1–R3

Table 3 shows the frequency of controlled drug identification across sample form factor in each region (photos shown in Figure 2 and Figure S9). In R1–R3, 81 of the 89 (91.0%) SC positive samples were for RF e‐cigarettes/e‐liquids [3 (3.4%) LB, 36 (40.4%) UB and 42 (47.2%) RF] [Figure 3(b)]. The remaining eight (9.0%) were SU, with one of these from R1 and seven from R2 (6 from a single school R2.S1). Seven of the eight SC positive SU contain an illegally (in the United Kingdom) large amount of e‐liquid (>2 mL).

There were 170 items in total for the refillable form factor, with 47.6% containing a SC (81/170), whereas only 2.3% of 343 SU were positive for a SC (8/343). THC containing e‐cigarettes were evenly split with two SU and three RF. These THC products are commercially available, the SU appear to have been imported and the RF are a device marketed for THC oil/resin (Figure S9).

A breakdown of SC concentration for each form factor in R1 and R2 is shown in Table 4. RF e‐cigarettes/e‐liquids had a higher median concentration than found in SU examples [0.56 (IQR = 1.03) and 0.16 (IQR = 0.13) mg mL^−1^, respectively]. E‐liquid (LB and UB) had a median concentration of 1.24 (IQR = 0.89) mg mL^−1^ compared to 0.49 (IQR = 0.84) mg mL^−1^ for RF.

The ‘typical’ colour of e‐liquid in SC negative branded e‐cigarettes was predominantly yellow (73.7%), brown (17%) and clear (8.1%). Other colours observed were green (0.8%) and orange (0.4%). SC positive e‐cigarettes also had a high number of ‘typical’ e‐liquid colours (60.2% yellow, brown and clear), but also had 39.8% ‘atypical’ colours (e.g. green, blue and pink) [Figure 3(c)].

### GC–MS results from R4 sampling

There were 50 suspicious e‐cigarettes/e‐liquids in total with 16 (32%) being positive for SC and 2 (4%) positive SU THC e‐cigarettes (data in Table S6 and photographs in Figure S10). In terms of physical presentation, 12 (75%) SC positives were in RF, three (19%) in UB, one (6%) in SU and none in LB. A total of 75% of samples contained MDMB‐4en‐PINACA and 25% containing 4F‐MDMB‐BINACA. Data from R4 mirror our findings from R1–R3 and increase the extent to which our findings may be generalizable to other schools in England.

### Correlation of school demographics with SC positive e‐cigarettes

Figure 3(d),(e) shows the correlation between the percentage of SC e‐cigarette presence versus social metrics from Table 2. There is potentially a positive (linear) correlation between the presence of SC e‐cigarettes and FSM, persistent absence and fraction of SEN support. A Pearson's correlation analysis confirms the fraction of FSM is positively correlated, r = 0.65 and *P* = 0.003. However, there is weak/no correlation with persistent absence (r = 0.30, *P* = 0.015), the percentage of SEN support (r = 0.50, *P* = 0.847) and EHCPs (r = 0.27, *P* = 0.007).

## DISCUSSION

Although it is illegal in the United Kingdom to sell nicotine inhaling products such as e‐cigarettes to under 18–year‐olds [26], it is prevalent in school age children [20]. SCs and THC are illegal in the United Kingdom. Compared to THC, SCs are estimated to have a 30‐fold greater risk of emergency medical treatment [5]. We report the first study to identify and quantify SCs in e‐cigarettes collected from schools, which shows an alarmingly high prevalence found in English schools. SC positive samples were identified in three quarters of schools and in all regions studied. The percentage of SC positive samples is consistent in the geographically distinct regions. These results should raise caution for schools across the country. In comparison, there was a relative lack of THC e‐cigarettes identified, which is inconsistent with a third of people under 17‐years‐old having used cannabis [27]. Indeed, since the 1980s, the social view of cannabis use is that it is increasingly an unremarkable feature of adolescent life [28].

How can these findings be reconciled? Our hypothesis is that young people are under the impression they are buying ‘cannabis’, but are being sold SCs instead. Evidence from WEDINOS support this notion, with SC e‐cigarettes almost never being the purchase intent [17]. THC e‐cigarettes are relatively expensive (~£15–£65). This compares with as little as approximately £3 for 2 mL (a full e‐cigarette) of SC liquid [18]. Additionally, on‐line webstores market SCs as essentially interchangeable with cannabis and imply it is legal [18]. The lower price of SC, together with marketing and an assumed inability of consumers to determine their content, may explain the high prevalence of SCs. Moreover, we would argue this trend is relatively recent, given the media reports appear to have ‘peaked’ in the United Kingdom in 2023/2024 (the time of writing). Crucially, there are no studies examining the pharmacology of SCs in children, and therefore, the true risk to health in both the immediate and long term is unknown.

It is possible to examine e‐cigarettes/e‐liquids by eye to make predictions about which are likely to contain SCs or THC. UBs were mostly positive for SCs as were nearly half of RF. This tracks with the availability of SCs from web shops being predominantly via bottles of liquid [18]. SCs were rarely found in LBs (commercial packaging) or SU e‐cigarettes. We do not suggest these commercial products contained SCs at source. The colour of e‐liquid is also an indicator with blue, green, red, pink and purple colours essentially always being SC positive. Nearly all SC negative e‐liquids were yellow, clear or brown, but these colours should not be assumed to be negative as over half SC positives were also these colours. That SCs in SU e‐cigarettes are both relatively rare and ‘low’ concentration [Figure 3(a)] suggests that at present these are not the critical modality of concern. THC e‐cigarettes are specifically designed for THC oil/resin and have different forms to RFs used for SC liquid. The THC oil/resin is also clearly identifiable by eye and odour. Using these guidelines and training, a teacher or other responsible adult could, as a reasonable guess, establish the high probability of SCs or THC being present.

We were interested if the distribution in SC concentration reflected a particular sample type. Samples in the ‘low’ concentration range (below 0.15 mg mL^−1^) were dominated by RFs and SU e‐cigarettes, while 'high’ concentration samples (above 1.13 mg mL^−1^) are mostly e‐liquid bottles. Given almost all SC e‐cigarette liquids are sold on‐line as bottles of liquid [18], the concentration present in the bottles would seem the ‘intended’ concentration.

Comparison of the school characteristic data show the schools compared are largely similar to each other and the England average. We note that the fraction of FSM for R1–R3 [median, 29.4 (IQR = 22.7%)] is slightly higher than the English average [median, 25.7 (IQR = 19.9%)] at the time of writing, but the difference is not over large given the absolute magnitude of the values and size of IQR. Therefore, these regions are meaningfully comparable and representative of English schools. Our data shows a positive correlation of SC positive e‐cigarettes with FSM, a specific metric of social deprivation. This further highlights the potential importance of the low price of SCs when compared to THC in e‐cigarettes. We would point to the ample evidence that the harm arising from drugs becomes greater with metrics of social deprivation [29].

### Limitations

Although this article demonstrates the widespread occurrence of SC containing e‐cigarettes in schools from diverse geographic regions of England, the generalisability of the percentage of SC positive e‐cigarettes should be interpreted with caution because of the non‐probability sampling methodology and sample size. The SC positive samples are also nuanced by the presence of very low concentration refillable samples that are most likely a result of previous, rather than current SC use. The limited size of the study also meant only a small number of schools could be used to look for correlations with the school characteristics. However, the statistics did suggest an underlying trend with FSM. The current study did not link the SC samples with the number of young people that the samples were seized from.

## CONCLUSIONS

Our findings have immediate implications for harm reduction interventions that should be developed for schools, as well as the stark need to better understand the risk to the health of children when they consume SCs, both acute and longer term. There is also a need to expand this work to inform the targeting of intervention and establish whether the prevalence of SCs in e‐cigarettes is high across the wider United Kingdom region or if this phenomenon is geographically or socially patterned.

Finally, our findings are immediately applicable to policy, demonstrating the imminent banning of SU e‐cigarettes in the United Kingdom [30] will not meaningfully affect the presence of SCs in schools, precisely because they are relatively rare in single use e‐cigarettes. An unintended consequence could be an increase in the use of refillable devices and the potential exposure to SCs within e‐cigarette liquids. This intelligence is further useful for policing efforts in the community and to identify modes of supply. We highlight that education can be an effective tool in combating drug‐related harm, particularly where young people are involved and well informed.

## AUTHOR CONTRIBUTIONS

**Gyles E. Cozier:** Conceptualization (equal); data curation (equal); formal analysis (equal); investigation (equal); methodology (equal); project administration (equal); writing—original draft (equal); writing—review and editing. **Matthew Gardner:** Conceptualization (equal); data curation (equal); formal analysis (equal); investigation (equal); methodology (equal); project administration (equal); writing—original draft (equal); writing—review and editing (equal). **Sam Craft:** Conceptualization (equal); formal analysis (supporting); methodology (supporting); writing—original draft (supporting); writing—review and editing (supporting). **Martine Skumlien:** Conceptualization (equal); formal analysis (supporting); writing—original draft (supporting); writing—review and editing (supporting). **Jack Spicer:** Conceptualization (supporting); formal analysis (supporting); writing—original draft (supporting); writing—review and editing (supporting). **Rachael Andrews:** Formal analysis (supporting); investigation (supporting); writing—original draft (supporting); writing—review and editing (supporting). **Alexander Power:** Formal analysis (supporting); software (supporting); writing—original draft (supporting); writing—review and editing (supporting). **Tom Haines:** Funding acquisition (supporting); software (supporting); supervision (supporting); writing—original draft (supporting); writing—review and editing (supporting). **Richard Bowman:** Funding acquisition (supporting); methodology (supporting); supervision (supporting); writing—original draft (supporting); writing—review and editing (supporting). **Amy E. Manley:** Conceptualization (supporting); formal analysis (supporting); funding acquisition (supporting); methodology (supporting); writing—original draft (supporting); writing—review and editing (supporting). **Peter Sunderland:** Formal analysis (supporting); methodology (supporting); writing—original draft (supporting); writing—review and editing (supporting).**Oliver B. Sutcliffe:** Formal analysis (supporting); funding acquisition (supporting); methodology (supporting); writing—original draft (supporting); writing—review and editing (supporting). **Stephen M. Husbands:** Conceptualization (supporting); formal analysis (supporting); funding acquisition (supporting); methodology (supporting); supervision (supporting); writing—original draft (supporting); writing—review and editing (supporting). **Lindsey Hines:** Formal analysis (supporting); methodology (supporting); writing—original draft (supporting); writing—review and editing (supporting). **Gillian Taylor:** Conceptualization (equal); data curation (equal); formal analysis (equal); funding acquisition (equal); investigation (equal); methodology (equal); writing—original draft (equal); writing—review and editing. **Tom P. Freeman:** Conceptualization (supporting); formal analysis (supporting); funding acquisition (supporting); methodology (supporting); project administration (supporting); supervision (supporting); writing—original draft (supporting); writing—review and editing (supporting). **Jennifer Scott:** Conceptualization (supporting); formal analysis (supporting); funding acquisition (supporting); methodology (supporting); project administration (supporting); supervision (supporting); writing—original draft (supporting); writing—review and editing (supporting). **Christopher R. Pudney:** Conceptualization (lead); data curation (lead); formal analysis (supporting); funding acquisition (lead); investigation (supporting); methodology (supporting); project administration (lead); software (lead); supervision (lead); writing—original draft (lead); writing—review and editing (lead).

## DECLARATION OF INTERESTS

None.

## Supporting information


**Table S1.** Media reports of adverse effects in schools associated with vaping and putatively associated with SC use.
**Table S2.** Results summary for all samples in R1.
**Table S3.** Results summary for all samples in R2.
**Table S4.** Results summary for all samples in R3.
**Table S5.** Results summary for all samples in R4.
**Table S6.** Data summary for R1–‐3.
**Table S7.** LC‐–MS parent molecule and fragment mass to charge (mz) ratios used to confirm compounds found in the e‐cigarettes/liquids.
**Figure S1.** Example LC‐–MS chromatogram and spectra for sample R1S1.2 with MDMB‐4en‐PINACA, 4F‐MDMB‐BINACA and MDMB‐INACA SCs identified.
**Figure S2.** Example LC‐–MS chromatogram and spectra for sample R1S12.119 with ADB‐BUTINACA identified.
**Figure S3.** Structures of illicit drugs identified.
**Figure S4.** Example 1H NMR spectra used for qNMR on sample R1S8.62 containing MDMB‐4en‐PINACA.
**Figure S5.** Example 1H NMR spectra used for qNMR on sample R2S1.3 containing MDMB‐4en‐PINACA.
**Figure S6.** Example 1H NMR spectra used for qNMR on sample R1S12.121 containing ADB‐BUTINACA.
**Figure S7.** Example 1H NMR spectra used for qNMR on sample R1S2.13 containing MDMB‐4en‐PINACA.
**Figure S8.** Plot showing the qNMR calculated values of a standard concentration range of MDMB‐4en‐PINACA in e‐cigarette liquid.
**Figure S9.** Photographs of THC e‐cigarettes from R1–‐3.
**Figure S10.** Photographs of SC and THC e‐cigarettes from R4.

## Data Availability

The data that support the findings of this study are available from the corresponding author upon reasonable request.
