# Supplementary material for: Synthetic cannabinoids in e‐cigarettes seized from English schools
Source: Addiction. 2025 Jun 26;120(10):1995–2004. doi: 10.1111/add.70110 (PMC12426342; doi:10.1111/add.70110)
Supplement: Supplementary file 1 — Table S1. Media reports of adverse effects in schools associated with vaping and putatively associated with SC use. Table S2. Results summary for all samples in R1. Table S3. Results summary for all samples in R2. Table S4. Results summary for all samples in R3. Table S5. Results summary for all samples in R4. Table S6. Data summary for R1–‐3. Table S7. LC‐–MS parent molecule and fragment mass to charge (mz) ratios used to confirm compounds found in the e‐cigarettes/liquids. Figure S1. Example LC‐–MS chromatogram and spectra for sample R1S1.2 with MDMB‐4en‐PINACA, 4F‐MDMB‐BINACA and MDMB‐INACA SCs identified. Figure S2. Example LC‐–MS chromatogram and spectra for sample R1S12.119 with ADB‐BUTINACA identified. Figure S3. Structures of illicit drugs identified. Figure S4. Example 1H NMR spectra used for qNMR on sample R1S8.62 containing MDMB‐4en‐PINACA. Figure S5. Example 1H NMR spectra used for qNMR on sample R2S1.3 containing MDMB‐4en‐PINACA. Figure S6. Example 1H NMR spectra used for qNMR on sample R1S12.121 containing ADB‐BUTINACA. Figure S7. Example 1H NMR spectra used for qNMR on sample R1S2.13 containing MDMB‐4en‐PINACA. Figure S8. Plot showing the qNMR calculated values of a standard concentration range of MDMB‐4en‐PINACA in e‐cigarette liquid. Figure S9. Photographs of THC e‐cigarettes from R1–‐3. Figure S10. Photographs of SC and THC e‐cigarettes from R4. [file ADD-120-1995-s001.docx]

*Supporting information -* Synthetic cannabinoids consumed via e-cigarettes in English schools

Gyles E. Cozier,^1‡^ Matthew Gardner,^1‡^ Sam Craft,^2^ Martine Skumlien,^2^ Jack Spicer,^3^ Rachael Andrews,^1^ Alexander Power,^4^ Tom Haines,^4^ Richard Bowman,^5^ Amy E. Manley,^6^ Peter Sunderland,^1^ Oliver B. Sutcliffe,^7^ Stephen M. Husbands,^1^ Lindsey Hines,^2^ Gillian Taylor,^8^ Tom P. Freeman,^2^* Jennifer Scott,^9^* Christopher R. Pudney^1^*

^1^Department of Life Sciences, ^2^Department of Psychology, ^3^Department of Social and Policy Science, ^4^Department of Computer Science, University of Bath, Bath, BA2 7AY, UK. ^5^School of Physics and Astronomy, University of Glasgow, Glasgow, G12 8QQ, UK. ^6^Bristol Medical School, University of Bristol, Bristol, BS8 2PS, UK. ^7^MANchester DRug Analysis & Knowledge Exchange (MANDRAKE), Department of Natural Sciences, Manchester Metropolitan University, Manchester, M1 5GD. ^8^School of Health and Life Sciences, Teesside University, Middlesbrough, TS1 3BX ^9^Centre for Academic Primary care, Bristol Medical School, University of Bristol, Bristol, BS8 2PS, UK.

Analytical methods

Table S1. Media reports of adverse effects in schools associated with vaping and putatively associated with SC use

Table S2. Results summary for all samples in R1

Table S3. Results summary for all samples in R2

Table S4. Results summary for all samples in R3

Table S5. Results summary for all samples in R4

Table S6. Data summary for R1-3

Table S7. LC-MS parent molecule and fragment mass to charge (mz) ratios used to confirm compounds found in the e-cigarettes/liquids

Figure S1. Example LC-MS chromatogram and spectra for sample R1S1.2 with MDMB-4en-PINACA, 4F-MDMB-BINACA and MDMB-INACA SCs identified

Figure S2. Example LC-MS chromatogram and spectra for sample R1S12.119 with ADB-BUTINACA identified

Figure S3. Structures of illicit drugs identified

Figure S4. Example 1H NMR spectra used for qNMR on sample R1S8.62 containing MDMB-4en-PINACA

Figure S5. Example 1H NMR spectra used for qNMR on sample R2S1.3 containing MDMB-4en-PINACA

Figure S6. Example 1H NMR spectra used for qNMR on sample R1S12.121 containing ADB-BUTINACA

Figure S7. Example 1H NMR spectra used for qNMR on sample R1S2.13 containing MDMB-4en-PINACA

Figure S8. Plot showing the qNMR calculated values of a standard concentration range of MDMB-4en-PINACA in e-cigarette liquid

Figure S9. Photographs of THC e-cigarettes from R1-3

Figure S10. Photographs of SC and THC e-cigarettes from R4

# ANALYTICAL METHODS

## LC-MS

For LC-QToF-MS analysis, all e-cigarette liquid samples were initially diluted 20 000x in HPLC grade ethanol, with repeats at higher concentration where needed/possible. Analyses were performed using an Agilent QToF 6545 with a Jetstream electrospray ionization (ESI) source coupled to an Agilent 1260 Infinity II Quaternary pump HPLC with a 1260 autosampler, column oven compartment, and variable wavelength detector (VWD). The mobile phases used were (A) LC-MS grade water with 0.1% formic acid and (B) acetonitrile with 0.1% formic acid. The gradient used was 95:5 A:B from 0.00-0.60 min, change to 0:100 A:B over 0.60-3.00 min, held at 0:100 A:B from 3.00-5.50 min, change to 95:5 A:B over 5.50-5.60 min, and held at 95:5 A:B from 5.6-7.6 min. The flow rate was 0.5 mL/min at 50ºC and 5 μL of the sample was injected onto an EC-C18 3.0 x 50 mm, 2.7 μm particle size column (InfinityLab Poroshell 120, Agilent Technologies). The MS was operated in positive ionization mode with the gas temperature at 250°C, the drying gas at 11 L/min, and the nebulizer gas at 35 psi (2.41 bar). The sheath gas temperature was set to 300ºC and the flow rate was 12 L/min. The MS was calibrated using a reference calibrant introduced from an independent ESI reference sprayer. The VCap, Fragmentor and Skimmer were set to 3500, 160, and 45 V, respectively. The MS was operated in all-ions mode with three collision energy scan segments at 0, 20, and 40 eV.

The VWD was set to detect at 280 nm wavelength at a frequency of 2.5 Hz. Data processing was automated in Qual 10, with the molecular feature extraction set to the largest 20 compounds for [M^+^H]^+^, [M-H]^-^, and [M+HCOO]^-^ ions. The results were also searched against the online mass spectral databases HighResNPS (containing over 2300 unique compound entries) and ForTox, with a forward score of 25 and reverse score of 70, and mass tolerances within 5 ppm of the reference library matches. Qualified ions had co-elution scores of ≥ 90, retention time tolerances of ± 0.10, and a minimum S/N of ≥ 5.00.

## GC-MS

Samples were diluted with methanol 50/50 ratio. GCMS analysis was preformed using a TQ-GC (Waters Corporation). Chromatographic separation was performed on a Thames Restek (DB-5, 30 m x 0.25 mm ID x 0.25 µm). The following temperature parameters were used: 60 °C (held for 3min) to 300 °C at 15 °C min-1, then held for 6 min. Injection port was 280°C, split ratio 25 mL/min. The analyser was set to scan m/z 50-500. GCMS peaks were identified through comparison to standards, NIST database and Cayman Chemicals forensic database.

## NMR

The quantitative nuclear magnetic resonance (NMR) method used was based on a study that quantified SCs in seized e-liquids.^18^ Samples were prepared by mixing 200 μL of e-cigarette liquid with 400 μL of methanol-d4 (MeOD) containing 3 mg of 3-(trimethylsilyl)propionic-2,2,3,3-d4 acid sodium salt (purity ≥ 99%, isotopic purity 98 atom % D) (TSP). A set of MDMB-4en-PINACA concentrations (10 – 0.1 mg/ml) were prepared in 50/50 propylene glycol/glycerol (PG/VG) as standard e-cigarette liquids to test the quantification method.

^1^H NMR data were recorded on a Bruker AvanceCore 400 MHz spectrometer (1H frequency of 400.130), with a zg pulse sequence composed of 3.18 s acquisition time, 128 scans and 20 s delay. Chemical shifts were referenced to 3.31 ppm for residual CD_2_HOD solvent peak (from MeOD) and are reported in ppm. NMR spectra were processed with Mestralab Mnova 14.1 using automatic phase and Whittaker smoother baseline corrections, followed by zero filling (4 x original size) and line broadening (1 Hz) to improve signal/noise ratio. Due to the large amounts of PG/VG in e-cigarette liquid, only the 4 peaks from the aromatic indazole core of the SCs could be reliably integrated. As many of these were used for the qNMR calculation, when not obscured by other additives in the e-cigarette liquid.

The following equation was used for the ^1^H qNMR quantitation:

$$[x] = \frac{n_{IC}\cdot{Int}_{x}\cdot{MW}_{x}\cdot m_{IC}}{n_{x}\cdot{Int}_{IC}{\cdot MW}_{IC}\cdot V_{s}}{\cdot P}_{IC}$$

Where [ ] denotes concentration in mg/mL, P is the purity, n is the number of protons, Int is the integral value, MW is the molecular weight, m is the mass in mg, V is the volume in mL, IC is the internal calibrant, x is the analyte, and s is the sample. As the indazole peaks of the different SC compounds overlayed, when multiple SC compounds were present in each sample, the molecular weight of the main SC, as judged by the LC-MS chromatogram, was used in the calculation.

| **Date** | **Location** | **Link** |
| --- | --- | --- |
| 30/04/2024 | West midlands | https://www.dailystar.co.uk/news/latest-news/deadly-spice-laced-vapes-leave-32709640 |
| 25/04/2024 | West Midlands | Email |
| 08/04/2024 | Belfast | https://www.belfasttelegraph.co.uk/news/education/surge-in-use-of-danger-drug-spice-in-vapes-as-ni-schools-warn-parents-of-concerns/a1420855242.html |
| 20/03/2024 | Kent | https://www.dailymail.co.uk/news/article-13218931/schoolboy-vape-laced-cannabis-spice-collapsed-hospital.html |
| 13/03/2024 | Teeside | https://www.gazettelive.co.uk/news/teesside-news/school-issues-warning-parents-after-28805683 |
| 01/03/2024 | Bolton | https://www.bbc.co.uk/news/uk-england-manchester-68536562 |
| 12/02/2024 | London | https://www.standard.co.uk/news/health/what-zombie-vapes-e-cig-children-spice-symptoms-b1138635.html |
| 19/01/2024 | Cleveland | https://www-gazettelive-co-uk.cdn.ampproject.org/v/s/www.gazettelive.co.uk/news/teesside-news/15-year-old-arrested-over-28480443.amp?amp_gsa=1&amp_js_v=a9&usqp=mq331AQIUAKwASCAAgM%3D#amp_tf=From%20%251%24s&aoh=17061028470033&referrer=https%3A%2F%2Fwww.google.com&ampshare=https%3A%2F%2Fwww.gazettelive.co.uk%2Fnews%2Fteesside-news%2F15-year-old-arrested-over-28480443 |
| 24/01/2024 | Lancaster | Multiple |
| 01/01/2024 | Plymouth | Email |
| 11-Dec-23 | Gwent | https://www.mirror.co.uk/news/uk-news/urgent-warning-parents-after-three-31650064 |
| 20-Dec-23 | Falkirk | https://www.dailymail.co.uk/news/article-12885841/boy-hospital-sick-vaping-spice-drug-school.html |
| 12-Nov-23 | Oldham | https://www.bbc.co.uk/news/health-67349297 |
| 01-Nov-23 | Sandwell | Email |
| 27-Oct-23 | Middlesborough | https://www.itv.com/news/tyne-tees/2023-10-27/children-collapsing-in-school-after-using-vapes-laced-with-spice |
| 25-Oct-23 | Selby | https://www.yorkpress.co.uk/news/23877890.selby-police-warn-reports-vapes-containing-drug-spice/ |
| 12-Jul-23 | Oldham | https://www.oasisacademyoldham.org/news-and-events/latest-news/news-post-page/~board/oldham-news/post/oldham-vape-warning-reminder-for-parents-and-carers |

**Table S1. Media reports of adverse effects in schools associated with vaping and putatively associated with SC use**

Table S2. Results summary for all samples in R1

| R1S1 | Sample type | Detail | Liquid colour | LC-MS result | Quant (mg / mL) |
| --- | --- | --- | --- | --- | --- |
| 1 | RF | Vaporesso | Orange | NDD | - |
| 2 | RF | Vaporesso | Yellow | MDMB-4en-PINACA  4F-MDMB-BINACA  MDMB-INACA | 0.63 |
| 3 | LB | 'Elux Mr Blue', Yellow Liquid | Yellow | NDD | - |
| 4 | LB | 'Elux Lemon and Lime', Yellow Liquid | Yellow | NDD | - |
| 5 | RF | - | - | MDMB-4en-PINACA | IM |
| 6 | RF | Vaporesso | Brown | NDD | - |
| 7 | RF | Vaporesso | Green | MDMB-4en-PINACA  4F-MDMB-BINACA  MDMB-INACA | 0.14 |
| 8 | RF | Vaporesso | Yellow | MDMB-4en-PINACA  4F-MDMB-BINACA  MDMB-INACA | IM |
| 9 | UB | - | Yellow | MDMB-4en-PINACA  4F-MDMB-BINACA  MDMB-INACA | 0.89 |
| R1S2 | Sample type | Detail | Liquid colour | LC-MS result | Quant (mg / mL) |
| 10 | RF | Vaporesso | Clear | MDMB-4en-PINACA  MDMB-INACA | 0.11 |
| 11 | SU | - | - | NT | - |
| 12 | SU | Gold bar | Yellow | NDD | - |
| 13 | LB | 'Elux Mr Blue', Yellow Liquid | Blue | MDMB-4en-PINACA  MDMB-PINACA | 2.69 |
| 14 | RF | Vaporesso | Green | MDMB-4en-PINACA  MDMB-INACA | 1.32 |
| R1S3 | Sample type | Detail | Liquid colour | LC-MS result | Quant (mg / mL) |
| 15 | SU | Crystal bar, Cherry fizz | - | NT | - |
| R1S4 | Sample type | Detail | Liquid colour | LC-MS result | Quant (mg / mL) |
| 16 | SU | Enjoy Ultra | - | NT | - |
| 17 | SU | OXAV | - | NT | - |
| 18 | RF | Vaporesso, orange liquid | Yellow | MDMB-4en-PINACA  MDMB-INACA  4F-MDMB-BINACA | 0.56 |
| 19 | SU | Hit Fusion, Pink Lemonade | - | NT | - |
| 20 | SU | Elf Bar | - | NDD | - |
| R1S5 | Sample type | Detail | Liquid colour | LC-MS result | Quant (mg / mL) |
| 21 | SU | Crystal Bar, Pineapple peach mango | - | NT | - |
| 22 | SU | Crystal Bar, Cola ice | - | NT | - |
| 23 | SU | Crystal bar, Cola ice | - | NT | - |
| R1S6 | Sample type | Detail | Liquid colour | LC-MS result | Quant (mg / mL) |
| 24 | SU | Randm Tornado | - | NT | - |
| 25 | SU | Sky Hunter | - | NT | - |
| 26 | RF | Vaporesso | Brown | MDMB-4en-PINACA | 0.29 |
| 27 | UB | - | Yellow | MDMB-4en-PINACA  4F-MDMB-BINACA | 0.51 |
| R1S7 | Sample type | Detail | Liquid colour | LC-MS result | Quant (mg / mL) |
| 28 | SU | Lost Mary, Blue Razz Cherry | - | NDD | - |
| 29 | SU | Crystal Bar, Blueberry sour raspberry | - | NT | - |
| 30 | SU | Crystal Bar, Blueberry peach ice | - | NDD | - |
| 31 | SU | Twister bar, Strawberry raspberry cherry | - | NDD | - |
| 32 | SU | Elfbar | - | NDD | - |
| 33 | SU | Lost Mary, Pineapple ice | - | NDD | - |
| 34 | SU | Lost Mary, Cherry ice | - | NT | - |
| 35 | SU | Lost Mary, Cherry ice | - | NDD | - |
| 36 | SU | Elfbar, Cola | - | NDD | - |
| 37 | SU | Brand unknown | - | NT | - |
| 38 | SU | Crystal Bar, Lemon and Lime | - | NDD | - |
| 39 | SU | Elfbar | - | NDD | - |
| 40 | SU | Lost Mary, Pineapple ice | - | NT | - |
| 41 | SU | Crystal Bar, Tiger blood | - | NDD | - |
| 42 | SU | Crystal bar, Lemon and Lime | - | NDD | - |
| 43 | SU | Lost Mary, Pineapple ice | - | NDD | - |
| 44 | SU | Elfbar | - | NDD | - |
| 45 | SU | Lost Mary, Double Apple | - | NT | - |
| 46 | SU | Lost Mary, Blue Razz ice | - | NT | - |
| 47 | SU | Lost Mary, Kiwi passion fruit guava | - | NDD | - |
| 48 | SU | Bloody Mary Crystal, Strawberry ice | - | NT | - |
| 49 | SU | Crystal Bar, Lemon and Lime | - | NT | - |
| 50 | RF | Vaporesso | Yellow | MDMB-4en-PINACA | IM |
| 51 | SU | Crystal Bar, Lemon and Lime | - | NT | - |
| 52 | RF | Vaporesso | Green | ADB-BUTINACA  MDMB-4en-PINACA | 2.04 |
| 53 | RF | Vaporesso | Yellow | MDMB-4en-PINACA | ‘low’ |
| R1S8 | Sample type | Detail | Liquid colour | LC-MS result | Quant (mg / mL) |
| 54 | SU | Unknown brand, Cherry ice | Yellow | NDD | - |
| 55 | LB | Labelled bottle, Elux, Blueberry raspberry, yellow liquid | Yellow | NDD | - |
| 56 | RF | Vaporesso, yellow liquid | Yellow | MDMB-4en-PINACA | ‘low’ |
| 57 | RF | Vaporesso, yellow liquid | Yellow | NDD | - |
| 58 | RF | Vaporesso, Brown liquid | Brown | NDD | - |
| 59 | LB | Labelled bottle, Frunk, Pineapple express, Clear liquid | Clear | NDD | - |
| 60 | LB | Labelled bottle, Elux, MR Blue, Green liquid | Green | MDMB-4en-PINACA | ‘low’ |
| 61 | LB | Labelled bottle, Elux, Cherry ice, yellow liquid | Yellow | NDD | - |
| 62 | UB | - | Clear | MDMB-4en-PINACA | 3.52 |
| 63 | RF | Vaporesso, orange liquid | Yellow | MDMB-4en-PINACA | ‘low’ |
| 64 | LB | Labelled bottle, Elux, Blueberry cherry cranberry, orange liquid | Yellow | MDMB-4en-PINACA | ‘low’ |
| 65 | RF | Vaporesso, green liquid | Green | MDMB-4en-PINACA | 1.31 |
| 66 | SU | Crystal bar, Cherry ice |  | NT | - |
| 67 | SU | Crystal bar, Watermelon strawberry | - | NT | - |
| 68 | SU | Crystal bar, Lemon and Lime | - | NT | - |
| 69 | SU | Crystal bar, Cherry ice | Yellow | NDD | - |
| 70 | RF | Vaporesso, yellow liquid | Yellow | MDMB-4en-PINACA | 0.37 |
| 71 | RF | Vaporesso, green liquid | Green | MDMB-4en-PINACA  ADB-4en-PINACA | 0.39 |
| 72 | SU | Randm tornado | Yellow | MDMB-4en-PINACA | IM |
| 73 | RF | Vaporesso, green liquid | Green | NDD | - |
| 74 | SU | R and M, Magic mint | - | NDD | - |
| 75 | RF | Arc Mini | - | NT | - |
| 76 | SU | Vuse Go | - | NT | - |
| 77 | SU | Crystal Bar, Sour apple blueberry | - | NDD | - |
| 78 | SU | Crystal Bar, Fizzy cherry | - | NDD | - |
| 79 | RF | Vaporesso | Yellow | MDMB-4en-PINACA | ‘low’ |
| 80 | SU | Bloody Mary, triple berry | Yellow | NDD | - |
| 81 | SU | Crystal bar, Lemon and Lime | - | NT | - |
| 82 | SU | Crystal bar, Lemon and Lime | Yellow | NDD | - |
| 83 | LB | Labelled bottle, Elflic, strawberry ice cream, yellow liquid | Yellow | NDD | - |
| 84 | SU | Elfbar, Strawberry raspberry cherry ice | Yellow | NDD | - |
| 85 | UB | Unlabelled bottle, clear liquid | Clear | NDD | - |
| 86 | SU | Zillion | Yellow | NDD | - |
| 87 | SU | Elfbar, watermelon | Yellow | NDD | - |
| 88 | LB | Zeus, clear liquid | Clear | NDD | - |
| 89 | LB | Zeus, clear liquid | Clear | NDD | - |
| R1S9 | Sample type | Detail | Liquid colour | LC-MS result | Quant (mg / mL) |
| 90 | RF | Vaporesso | Green | MDMB-4en-PINACA | 0.42 |
| 91 | RF | Vaporesso | Yellow | NDD | - |
| 92 | RF | Vaporesso | Green | MDMB-4en-PINACA | 0.36 |
| 93 | UB | - | Blue | MDMB-4en-PINACA | 2.6 |
| 94 | UB | - | Blue | MDMB-4en-PINACA MDMB-PINACA | 1.24 |
| 95 | SU | Enjoy Ultra, peach watermelon strawberry | - | NDD | - |
| R1S10 | Sample type | Detail | Liquid colour | LC-MS result | Quant (mg / mL) |
| 96 | SU | Crystal bar | Yellow | NDD | - |
| 97 | SU | Elfbar | Yellow | NDD | - |
| 98 | SU | Gold bar | Yellow | NDD | - |
| 99 | SU | Crystal bar | Yellow | NDD | - |
| 100 | SU | Crystal bar | Yellow | NDD | - |
| 101 | SU | Ene Legend | Yellow | NT | - |
| 102 | SU | Hyati pro | Yellow | NDD | - |
| 103 | SU | Elux legend pro | Yellow | NDD | - |
| 104 | SU | Elfbar | Brown | NDD | - |
| 105 | SU | Crystal bar | Yellow | NT | - |
| 106 | SU | Crystal bar | Yellow | NT | - |
| 107 | SU | Elfbar | Brown | NDD | - |
| 108 | SU | Randm Tornado | Yellow | NDD | - |
| 109 | SU | Elfbar | Yellow | NDD | - |
| 110 | SU | Hyati pro | Yellow | NDD | - |
| 111 | SU | Lost mary | Yellow | NT | - |
| 112 | SU | Gold bar | Yellow | NDD | - |
| 113 | SU | Crystal bar | Yellow | NT | - |
| 114 | SU | - | Yellow | NDD | - |
| 115 | SU | Ene Legend | Brown | NDD | - |
| 116 | SU | - | Brown | NDD | - |
| R1S11 | Sample type | Detail | Liquid colour | LC-MS result | Quant (mg / mL) |
| 117 | UB | - | Pink | MDMB-4en-PINACA  MDMB-PINACA | 1.5 |
| R1S12 | Sample type | Detail | Liquid colour | LC-MS result | Quant (mg / mL) |
| 118 | RF | Vaporesso | Red | MDMB-4en-PINACA | ‘low’ |
| 119 | UB | - | Purple | ADB-BUTINACA | 2.16 |
| 120 | UB | - | Yellow | ADB-BUTINACA | 1.19 |
| 121 | UB | - | Yellow | ADB-BUTINACA | 1.28 |
| 122 | UB | - | Green | MDMB-4en-PINACA  4F-MDMB-BINACA  MDMB-INACA | 1.28 |
| 123 | UB | - | Green | MDMB-4en-PINACA  4F-MDMB-BINACA  MDMB-INACA | 0.34 |
| 124 | SU | - | Brown resin | THC | 508.15 |
| 125 | SU | Crystal bar | - | NT | - |
| 126 | SU | Crystal bar | - | NT | - |
| 127 | SU | Enjoy ultra | - | NT | - |
| 128 | SU | Twins maxfel | - | NT | - |
| 129 | SU | Lost mary | - | NT | - |
| 130 | SU | Enjoy ultra | - | NT | - |
| 131 | SU | 88. vapes | - | NT | - |
| 132 | SU | Hayati pro ultra | - | NT | - |
| 133 | SU | Crystal bar | - | NT | - |
| 134 | SU | Randm tornado | - | NT | - |
| 135 | SU | Randm tornado | - | NT | - |
| 136 | SU | Randm tornado | - | NT | - |
| 137 | SU | 88 vapes | - | NT | - |
| 138 | SU | Crystal original | - | NT | - |
| 139 | SU | Lost mary | - | NT | - |
| 140 | SU | Lost mary | - | NT | - |
| 141 | SU | Randm tornado | - | NT | - |
| 142 | SU | Insta bar | - | NT | - |
| 143 | SU | 88. vapes | - | NT | - |
| 144 | SU | - | - | NT | - |
| 145 | SU | - | - | NT | - |
| 146 | SU | - | - | NT | - |
| 147 | SU | - | - | NT | - |
| 148 | SU | - | - | NT | - |
| 149 | SU | - | - | NT | - |
| 150 | SU | - | - | NT | - |
| R1S13 | Sample type | Detail | Liquid colour | LC-MS result | Quant (mg / mL) |
| 151 | SU | Twister bar | - | NT | - |
| 152 | SU | Crystal bar | Yellow | NDD | - |
| 153 | SU | Crystal bar | Yellow | NDD | - |
| 154 | SU | Crystal bar | - | NT | - |
| 155 | SU | Crystal bar | - | NT | - |
| 156 | SU | - | - | NT | - |

NDD, no drug detected; ‘low’, concentration below level of NMR measurement; IM, insufficient material; NT, not tested; SU, single use; RF, refillable; UB, unlabelled bottle; LB, labelled bottle.

Table S3. Results summary for all samples in R2

| R2S1 | Sample type | Detail | Liquid colour | LC-MS result | Quant (mg / mL) |
| --- | --- | --- | --- | --- | --- |
| 1 | UB | - | Purple | MDMB-4en-PINACA  MDMB-PINACA | 1.71 |
| 2 | UB | - | Clear | MDMB-4en-PINACA | IM |
| 3 | UB | - | Purple | MDMB-4en-PINACA  MDMB-PINACA | 1.56 |
| 4 | UB | - | Blue | MDMB-4en-PINACA | 2.92 |
| 5 | RF | Vaporesso | Clear | MDMB-4en-PINACA | IM |
| 6 | UB | - | Yellow | MDMB-4en-PINACA | IM |
| 7 | LB | Elux, Mr Blue | Yellow | NDD | - |
| 8a | SU | Hyati (2^nd^ pad) | Green | MDMB-4en-PINACA | 0.17 |
| 8b | SU | Hyati (main pad) | Yellow | MDMB-4en-PINACA | 0.05 |
| 9 | SU | - | Yellow | NDD | - |
| 10 | SU | - | Yellow | NDD | - |
| 11 | SU | - | Yellow | NDD | - |
| 12 | SU | - | Yellow | NDD | - |
| 13 | SU | - | Yellow | NDD | - |
| 14 | SU | - | Yellow | NDD | - |
| 15 | SU | - | Yellow | NDD | - |
| 16 | SU | - | Yellow | NDD | - |
| 17a | SU | Hyati (2^nd^ pad) | Purple | MDMB-4en-PINACA | 0.4 |
| 17b | SU | Hyati (main pad) | Brown | MDMB-4en-PINACA | IM |
| 18 | SU | - | Brown | NDD | - |
| 19 | SU | - | - | NT | - |
| 20 | SU | - | - | NT | - |
| 21 | SU | - | - | NT | - |
| 22 | SU | Randm Tornado | Yellow | MDMB-4en-PINACA  MDMB-INACA | IM |
| 23 | SU | Randm tornado | Green | MDMB-4en-PINACA | 0.3 |
| 24 | SU | - | Clear | NDD | - |
| 25 | SU | Quoa | Yellow | MDMB-4en-PINACA | 0.14 |
| 26 | SU | - | Yellow | NDD | - |
| 27 | SU | - | - | NT | - |
| 28 | SU | - | - | NT | - |
| 29 | SU | Hyati pro ultra | Yellow | MDMB-4en-PINACA | 0.15 |
| 30 | SU | - | Yellow | NDD | - |
| 31 | SU | - | Yellow | NDD | - |
| 32 | SU | - | Yellow | NDD | - |
| 33 | SU | - | Yellow | NDD | - |
| R2S2 | Sample type | Detail | Liquid colour | LC-MS result | Quant (mg / mL) |
| 34 | RF | - | - | NT | - |
| 35 | RF | Elfbar | Yellow | NDD | - |
| 36 | UB | - | Pink | Heroin  MDMB-4en-PINACA  ADB-BUTINACA | 0.1 (heroin), 0.28 (SC) |
| 37 | RF | 88 Vapes | Blue | MDMB-4en-PINACA | 0.4 |
| 38 | RF | Elfbar | Yellow | NDD | - |
| 39 | RF | Caliburn | Clear | NDD | - |
| 40 | LB | IVG salt, Cherry bubblegum breeze | Clear | NDD | - |
| 41 | UB | - | Clear | NDD | - |
| 42 | LB | Bar juice, watermelon | Yellow | NDD | - |
| 43 | LB | Elux | Yellow | NDD | - |
| 44 | RF | Vaporesso | Yellow | NDD | - |
| 45 | RF | RPM | Brown | MDMB-4en-PINACA  MDMB-INACA | ‘low’ |
| 46 | SU | Elux legend pro | Brown | NDD | - |
| 47 | SU | Elux legend pro | Brown | NDD | - |
| 48 | SU | Elux legend pro | Brown | NDD | - |
| 49 | SU | Elux legend pro | Brown | NDD | - |
| 50 | SU | Randm tornado | Brown | NDD | - |
| 51 | SU | Randm tornado | Yellow | NDD | - |
| 52 | SU | Randm tornado | Yellow | NDD | - |
| 53 | SU | Ene legend | Yellow | NDD | - |
| 54 | SU | Hyati pro ultra | Yellow | NDD | - |
| 55 | SU | Ene legend | Yellow | NDD | - |
| 56 | SU | Elfbar | Yellow | NDD | - |
| 57 | SU | Elfbar | Brown | NDD | - |
| 58 | SU | Elfbar | Brown | NDD | - |
| 59 | SU | Elfbar | Brown | NDD | - |
| 60 | SU | Elfbar | Yellow | NDD | - |
| 61 | SU | - | Brown | NDD | - |
| 62 | SU | Hyati | Yellow | NDD | - |
| 63 | SU | Hyati pro ultra | Yellow | NDD | - |
| 64 | SU | Lost Mary | Yellow | NDD | - |
| 65 | SU | Lost MAry | Brown | NDD | - |
| R2S3 | Sample type | Detail | Liquid colour | LC-MS result | Quant (mg / mL) |
| 66 | UB | - | Yellow | NDD | - |
| 67 | UB | - | Red | MDMB-4en-PINACA  MDMB-INACA | 0.54 |
| 68 | UB | - | Clear | MDMB-4en-PINACA | IM |
| 69 | UB | - | Yellow opaque | NDD | - |
| 70 | UB | - | Yellow | MDMB-BUTINACA  MDMB-4en-PINACA  MDMB-PINACA | 1.12 |
| 71 | UB | - | Yellow | MDMB-BUTINACA  MDMB-INACA | 0.97 |
| 72 | UB | - | Clear | MDMB-4en-PINACA  MDMB-INACA | 0.38 |
| 73 | UB | - | Clear | MDMB-4en-PINACA | IM |
| 74 | UB | - | Clear | MDMB-4en-PINACA | IM |
| 75 | RF | 88 Vapes | - | NT | - |
| 76 | RF | 88 Vapes | Yellow | MDMB-4en-PINACA | ‘low’ |
| 77 | SU |  |  | NT | - |
| 78 | RF | Vaporesso | Yellow | MDMB-4en-PINACA  ADB-4en-PINACA  MDMB-INACA | 1.26 |
| 79 | SU | WGA pro | - | NT | - |
| 80 | LB | - | Clear | NDD | - |
| 81 | LB | - | Yellow | NDD | - |
| 82 | LB | - | Yellow | NDD | - |
| 83 | LB | - | Yellow | NDD | - |
| 84 | LB | - | Yellow | NDD | - |
| 85 | LB | - | Yellow | NDD | - |
| 86 | LB | - | Yellow | NDD | - |
| 87 | RF | Vaporesso | Yellow | NDD | - |
| 88 | RF | Solus | Clear | MDMB-4en-PINACA | IM |
| 89 | RF | KRT | - | NDD | - |
| 90 | RF | Caliburn | Clear | NDD | - |
| 91 | RF | OXVA | Brown | MDMB-4en-PINACA  MDMB-BUTINACA  MDMB-INACA | 0.27 |
| 92 | RF | Vaporesso | Brown | MDMB-4en-PINACA | ‘low’ |
| 93 | RF | Just | - | NDD | - |
| 94 | RF | - | Brown | NDD | - |
| 95 | SU | Hyati pro | Yellow | NDD | - |
| 96 | SU | Hyati pro | Yellow | NDD | - |
| 97 | SU | Hyati pro | Yellow | NDD | - |
| 98 | SU | Hyati pro | Yellow | NDD | - |
| 99 | SU | Hyati pro | Yellow | NDD | - |
| 100 | SU | Hyati pro | Yellow | NDD | - |
| 101 | SU | Hyati pro | Yellow | NDD | - |
| 102 | SU | Hyati pro | Brown | NDD | - |
| 103 | SU | Hyati pro | Yellow | NDD | - |
| 104 | SU | Hyati pro | Yellow | NDD | - |
| 105 | SU | Hyati pro | Yellow | NDD | - |
| 106 | SU | Hyati pro | Yellow | NDD | - |
| 107 | SU | Hyati pro | Yellow | NDD | - |
| 108 | SU | Hyati pro | Yellow | NDD | - |
| 109 | SU | Hyati pro | Yellow | NDD | - |
| 110 | SU | Hyati pro | Yellow | NDD | - |
| 111 | SU | Hyati pro | Yellow | NDD | - |
| 112 | SU | Hyati pro | Yellow | NDD | - |
| 113 | SU | Hyati pro | Brown | NDD | - |
| 114 | SU | Hyati pro | Yellow | NDD | - |
| 115 | SU | Crystal pro | - | NT | - |
| 116 | SU | Crystal pro | Yellow | NDD | - |
| 117 | SU | Crystal pro | - | NT | - |
| 118 | SU | WGA | Brown | NDD | - |
| 119 | SU | Hyati pro ultra | Clear | NDD | - |
| 120 | SU | Crystal bar | - | NT | - |
| 121 | SU | Crystal bar | - | NT | - |
| 122 | SU | Crystal bar | Clear | NDD | - |
| 123 | SU | Crystal bar | Yellow | NDD | - |
| 124 | SU | Crystal bar | - | NT | - |
| 125 | SU | Elux legend | Yellow | NDD | - |
| 126 | SU | Elux legend | Brown | NDD | - |
| 127 | SU | Elux legend | Yellow | NDD | - |
| 128 | SU | Elux legend | Yellow | NDD | - |
| 129 | SU | Elux legend | Yellow | NDD | - |
| 130 | SU | Elux legend | Brown | NDD | - |
| 131 | SU | Elux legend | Yellow | NDD | - |
| 132 | SU | Elux legend | Brown | NDD | - |
| 133 | SU | Ene Legend | Yellow | NDD | - |
| 134 | SU | Ene Legend | Yellow | NDD | - |
| 135 | SU | Randm Tornado | Yellow | NDD | - |
| 136 | SU | Randm Tornado | Yellow | NDD | - |
| 137 | SU | Randm Tornado | Yellow | NDD | - |
| 138 | SU | Randm Tornado | Yellow | NDD | - |
| 139 | SU | Randm Tornado | Yellow | NDD | - |
| 140 | SU | Randm Tornado | Brown | NDD | - |
| 141 | SU | Randm Tornado | Brown | NDD | - |
| 142 | SU | Randm Tornado | Brown | NDD | - |
| 143 | SU | Ene Legend | Yellow | NDD | - |
| 144 | SU | Elux legend | Yellow | NDD | - |
| 145 | SU | Elux legend | Yellow | NDD | - |
| 146 | SU | Crystal bar | Yellow | NDD | - |
| 147 | SU | Crystal bar | - | NT | - |
| 148 | SU | Crystal bar | Yellow | NDD | - |
| 149 | SU | Crystal bar | Yellow | NDD | - |
| 150 | SU | Elfbar | Yellow | NDD | - |
| 151 | SU | Elfbar | Yellow | NDD | - |
| 152 | SU | Elfbar | Clear | NDD | - |
| 153 | SU | Elfbar | Yellow | NDD | - |
| 154 | SU | Elfbar | Yellow | NDD | - |
| 155 | SU | Elfbar | Brown | NDD | - |
| 156 | SU | Elfbar | Yellow | NT | - |
| 157 | SU | Elfbar | Yellow | NDD | - |
| 158 | SU | Elfbar | Yellow | NDD | - |
| 159 | SU | Veev | Clear | NDD | - |
| 160 | SU | Lost mary | Yellow | NT | - |
| 161 | SU | - | Yellow | NDD | - |
| 162 | SU | - | Yellow | NDD | - |
| 163 | SU | Geek | Yellow | NDD | - |
| 164 | SU | - | Yellow | NDD | - |
| R2S4 | Sample type | Detail | Liquid colour | LC-MS result | Quant (mg / mL) |
| 165 | UB | - | Yellow | MDMB-4en-PINACA | IM |
| 166 | RF | Vaporesso | Yellow | MDMB-4en-PINACA | IM |
| 167 | - | - | Yellow | NDD | - |
| R2S5 | Sample type | Detail | Liquid colour | LC-MS result | Quant (mg / mL) |
| 168 | RF | Bear+ Aspire | Yellow | NDD | - |
| 169 | LB | - | Yellow | NDD | - |
| 170 | RF | Smok Fortis | - | NDD | - |
| 171 | RF | OXVA | Brown | NDD | - |
| 172 | RF | Tri Box | Yellow | MDMB-4en-PINACA | IM |
| 173 | SU | Hyati pro ultra | Yellow | NDD | - |
| 174 | SU | Hyati pro | Yellow | NDD | - |
| 175 | SU | Hyati pro | Yellow | NDD | - |
| 176 | SU | Randm Tornado | Yellow | NDD | - |
| 177 | SU | Randm Tornado | Yellow | NDD | - |
| 178 | SU | Hyati pro | Yellow | NDD | - |
| 179 | SU | - | Yellow | NDD | - |
| 180 | SU | - | Yellow | NDD | - |
| 181 | SU | Hyati pro ultra | Yellow | NDD | - |
| 182 | SU | - | Yellow | NDD | - |
| 183 | SU | Elux legend pro | Yellow | NDD | - |
| 184 | SU | Crystal bar | Yellow | NDD | - |
| 185 | SU | Elux legend pro | Brown | NDD | - |
| 186 | SU | Crystal bar | Yellow | NDD | - |
| 187 | SU | Crystal bar | Yellow | NDD | - |
| 188 | SU | Crystal bar | Yellow | NDD | - |
| 189 | SU | Crystal bar | - | NT | - |
| 190 | SU | Crystal bar | Yellow | NDD | - |
| 191 | SU | Crystal bar | Yellow | NDD | - |
| 192 | SU |  |  | NT | - |
| R2S6 | Sample type | Detail | Liquid colour | LC-MS result | Quant (mg / mL) |
| 193 | UB | - | Pink | MDMB-4en-PINACA  MDMB-PINACA | 2.45 |
| 194 | RF | Gotek cart | Pink | MDMB-4en-PINACA  MDMB-PINACA | 2.08 |
| 195 | RF | Gotek cart | Green | MDMB-4en-PINACA  MDMB-PINACA | 1.04 |
| 196 | SU | Hyati pro | Yellow | NDD | - |
| 197 | SU | Hyati pro | Yellow | NDD | - |
| 198 | SU | Elux legend | Brown | NDD | - |
| R2S7 | Sample type | Detail | Liquid colour | LC-MS result | Quant (mg / mL) |
| 199 | LB | Elux Banana ice | Yellow | NDD | - |
| 200 | LB | Elux, gummy bear | Yellow | NDD | - |
| 201 | LB | Elux, gummy bear | Yellow | NDD | - |
| 202 | SU | Randm Tornado | Yellow | NDD | - |
| 203 | SU | Hyati duo mesh | Yellow | NDD | - |
| 204 | SU | - | Yellow | NDD | - |
| 205 | SU | Lost Mary | Yellow | NDD | - |
| R2S8 | Sample type | Detail | Liquid colour | LC-MS result | Quant (mg / mL) |
| 206 | RF | Vaporesso | Yellow | NDD | - |
| 207 | SU | Randm Tornado | Yellow | NDD | - |
| 208 | SU | Randm Tornado | Clear | NDD | - |
| 209 | SU | Randm Tornado | Yellow | NDD | - |
| 210 | SU | Hyati pro ultra | Yellow | NDD | - |
| 211 | SU | Hyati pro ultra | Yellow | NDD | - |
| 212 | SU | Crystal bar | Yellow | NDD | - |
| 213 | SU | Crystal bar | Yellow | NDD | - |
| 214 | SU | Crystal bar | Yellow | NDD | - |
| 215 | SU | Hyati pro max | Yellow | NDD | - |
| 216 | SU | Crystal bar | Yellow | NDD | - |
| 217 | SU | 88 vapes | Yellow | NDD | - |
| 218 | SU | - | Yellow | NDD | - |
| R2S9 | Sample type | Detail | Liquid colour | LC-MS result | Quant (mg / mL) |
| 219 | RF | X-Priv | Yellow | MDMB-4en-PINACA | IM |
| 220 | UB | - | Yellow opaque | MDMB-4en-PINACA  MDMB-INACA | 0.82 |
| 221 | RF | - | Brown | MDMB-4en-PINACA  MDMB-INACA | 0.55 |
| 222 | LB | Heizen blue | Yellow | NDD | - |
| 223 | LB | EPIQ | Yellow | NDD | - |
| 224 | LB | Sense by vapearts | Clear | NDD | - |
| 225 | LB | Sense by vapearts | Clear | NDD | - |
| 226 | LB | Vape district, Pineapple mango | Clear | NDD | - |
| 227 | RF | Smok | Clear | NDD | - |
| 228 | SU | Randm Tornado | Clear | NDD | - |
| 229 | RF | - | Brown | 4F-MDMB-BINACA | IM |
| 230 | RF | - | Green | MDMB-4en-PINACA  4F-MDMB-BINACA | IM |
| 231 | RF | - | Brown | NDD | - |
| 232 | SU | Hyati pro | Yellow | NDD | - |
| 233 | SU | Hyati pro | Yellow | NDD | - |
| 234 | SU | Hyati pro | Yellow | NDD | - |
| 235 | SU | Hyati pro | Yellow | NDD | - |
| 236 | SU | Hyati pro | Yellow | NDD | - |
| 237 | SU | Hyati pro | Yellow | NDD | - |
| 238 | SU | Hyati pro | Yellow | NDD | - |
| 239 | SU | Hyati pro | Yellow | NDD | - |
| 240 | SU | Hyati pro | Yellow | NDD | - |
| 241 | SU | Hyati pro | Yellow | NDD | - |
| 242 | SU | Hyati pro | Yellow | NDD | - |
| 243 | SU | Hyati pro | Yellow | NDD | - |
| 244 | SU | Hyati pro | Yellow | NDD | - |
| 245 | SU | Hyati pro | Yellow | NDD | - |
| 246 | SU | Hyati pro | Yellow | NDD | - |
| 247 | SU | Ene Legend | Brown | NDD | - |
| 248 | SU | Ene Legend | Yellow | NDD | - |
| 249 | SU | Elux Legend | Brown | NDD | - |
| 250 | SU | Elux Legend | Brown | NDD | - |
| 251 | SU | Elux Legend | Brown | NDD | - |
| 252 | SU | Crystal bar | Yellow | NDD | - |
| 253 | SU | Crystal bar | - | NT | - |
| 254 | SU | Crystal prime | Yellow | NDD | - |
| 255 | SU | Randm Tornado | - | NT | - |
| 256 | SU | Randm Tornado | Brown | MDMB-4en-PINACA | 0.05 |
| 257 | SU | Elfbar | Brown | NDD | - |
| 258 | SU | Elfbar | Yellow | NDD | - |
| 259 | SU | Elfbar | Brown | NDD | - |
| 260 | SU | Elfbar | Brown | NDD | - |
| 261 | SU | Elfbar | Brown | NDD | - |
| 262 | SU | Crystal bar | Yellow | NDD | - |
| 263 | SU | Crystal bar | Yellow | NDD | - |
| 264 | SU | Crystal bar | Yellow | NDD | - |
| 265 | SU | - | - | NT | - |
| 266 | SU | - | Brown | NDD | - |
| 267 | SU | Crystal pro | Brown | NDD | - |
| 268 | SU | WGA | Yellow | NDD | - |
| 269 | SU | WGA | - | NT | - |
| 270 | SU | Hyati pro ultra | Yellow | NDD | - |
| 271 | SU | Lost mary | Green | NDD | - |
| 272 | SU | Lost mary | Yellow | NDD | - |
|  |  |  |  |  |  |

NDD, no drug detected; ‘low’, concentration below level of NMR measurement; IM, insufficient material; NT, not tested; SU, single use; RF, refillable; UB, unlabelled bottle; LB, labelled bottle.

Table S4. Results summary for all samples in R3

| R3S1 | Sample type | Detail | Liquid colour | Presumptive result |  |
| --- | --- | --- | --- | --- | --- |
| 1 | SU | Boutiq, French Toast | Yellow resin | THC |  |
| 2 | SU | CCELL | Yellow resin | THC |  |
| 3 | SU | Bubbglegum Runtz | Yellow resin | THC |  |
| 4 | RF | Argus | Yellow | SC |  |
| 5 | SU | Crystal bar | - | NDD |  |
| 6 | RF | Gotek | - | NDD |  |
| 7 | RF | Sonder | - | NDD |  |
| 8 | RF | Vaporesso | - | NDD |  |
| 9 | RF | Vaporesso | - | NDD |  |
| 10 | RF | Vaporesso | - | NDD |  |
| 11 | SU | Crystal prime | - | NDD |  |
| 12 | SU | Crystal prime | - | NDD |  |
| 13 | SU | Hyati pro, blue razz cherry | - | NDD |  |
| 14 | LB | Hyati, berry lemonade | - | NDD |  |
| 15 | LB | Hyati, strawberry watermelon | - | NDD |  |
| R3S2 | Sample type | Detail | Liquid colour | Presumptive result |  |
| 16 | RF | Vaporesso | Yellow | SC |  |
| 17 | RF | Vaporesso | Blue | SC |  |
| 18 | UB | - | Blue | SC |  |
| 19 | UB | - | Blue | SC |  |
| 20 | UB | - | Blue | SC |  |
| 21 | RF | Gotek | - | NDD |  |
| 22 | SU | Elfbar | - | NDD |  |
| 23 | RF | Sonder | - | NDD |  |
| 24 | RF | Vaporesso | - | NDD |  |
| 25 | RF | Vaporesso | - | NDD |  |
| 26 | LB | Hit Liquid | - | NDD |  |
| 27 | LB | Elux, berry lemonade | - | NDD |  |
| 28 | LB | Elux, gummy bear | - | NDD |  |
| 29 | SU | Hyati, Cherry cola | - | NDD |  |
| 30 | SU | Hyati, blue soure raspberry | - | NDD |  |
| 31 | SU | Hyati, triple mango | - | NDD |  |
| 32 | SU | Lux pro, Mr blue | - | NDD |  |
| 33 | SU | Hyati, strawberry raspberry ice | - | NDD |  |
| 34 | SU | Hyati | - | NDD |  |
| 35 | SU | Crystal prime | - | NDD |  |
| 36 | SU | Crystal prime | - | NDD |  |
| 37 | SU | Hyati ultra, blue razz gummy bear | - | NDD |  |
| 38 | SU | Hyati duo, rainbow sherbert | - | NDD |  |
| 39 | SU | Crystal, juicy peach | - | NDD |  |
| 40 | SU | Crystal pro plus | - | NDD |  |
| 41 | SU | Hyati, mad blue | - | NDD |  |
| R3S3 | Sample type | Detail | Liquid colour | Presumptive result |  |
| 42 | UB | - | Clear | SC |  |
| 43 | UB | - | Clear | SC |  |
| 44 | UB | - | Pink | SC |  |
| 45 | UB | - | Yellow | SC |  |
| 46 | UB | - | Yellow | SC |  |
| 47 | UB | - | Yellow | SC |  |
| 48 | LB | Elux, gummy bear | - | NDD |  |
| R3S4 | Sample type | Detail | Liquid colour | Presumptive result |  |
| 49 | RF | Gotek | Green | SC |  |
| R3S5 | Sample type | Detail | Liquid colour | Presumptive result |  |
| 50 | RF | Backpackboyz | Yellow resin | THC |  |
| 51 | RF | CCELL | Yellow | THC |  |
| 52 | RF | Gotek | Green | SC |  |
| 53 | SU | Elfbar | - | NDD |  |
| 54 | RF | Vaporesso | - | NDD |  |
| 55 | LB | Gost red | - | NDD |  |
| 56 | LB | Elux, Gummey bear | - | NDD |  |
| 57 | LB | Bar juice, blueberry soure raspberry | - | NDD |  |
| 58 | LB | Pod salt nexus | - | NDD |  |
| 59 | RF | Vaporesso | - | NDD |  |
| 60 | RF | Vaporesso | - | NDD |  |
| 61 | RF | Gotek | - | NDD |  |
| 62 | RF | Gotek | - | NDD |  |
| 63 | RF | Powtop | - | NDD |  |
| 64 | RF | Vaporesso | - | NDD |  |
| 65 | RF | Vaporesso | - | NDD |  |
| 66 | RF | Gotek | - | NDD |  |
| 67 | RF | Gotek | - | NDD |  |
| 68 | RF | Gotek | - | NDD |  |
| 69 | RF | Gotek | - | NDD |  |
| 70 | SU | Randm Tornado | - | NDD |  |
| 71 | SU | Randm Tornado | - | NDD |  |
| 72 | SU | Randm Tornado | - | NDD |  |
| 73 | SU | Crystal bar | - | NDD |  |
| 74 | SU | Crystal bar | - | NDD |  |
| 75 | SU | Crystal bar | - | NDD |  |
| 76 | SU | Crystal bar | - | NDD |  |
| 77 | SU | Crystal prime | - | NDD |  |
| 78 | SU | Hyati pro, blue razz cherry | - | NDD |  |
| 79 | SU | Hyati pro, blue razz cherry | - | NDD |  |
| 80 | SU | Hyati pro ultra | - | NDD |  |
| 81 | SU | Hyati | - | NDD |  |
| 82 | SU | Hyati | - | NDD |  |
| 83 | SU | Hyati | - | NDD |  |
| 84 | SU | Elux | - | NDD |  |

NDD, no drug detected; SC, synthetic cannabinoid; THC, tetrahydrocannabinol; SU, single use; RF, refillable; UB, unlabelled bottle; LB, labelled bottle.

Table S5. Results summary for all samples in R4.

| R4S7 | Sample type | Detail | Liquid colour | GC-MS result |  |
| --- | --- | --- | --- | --- | --- |
| 1 | SU | crystal prime | - | NDD |  |
| 2 | SU | Jeeter | - | THC |  |
| 3 | RF | ox | - | 4F-MDMB-BINACA |  |
| 4 | SU | novo | - | 4F-MDMB-BINACA |  |
| 5 | SU | non refillable firerose | - | NDD |  |
| 6 | RF | red oxva | - | 4F-MDMB-BINACA |  |
| 7 | RF | blue oxva | - | 4F-MDMB-BINACA |  |
| 8 | SU | happy vibes | - | NDD |  |
| 9 | SU | green crystal prime | - | NDD |  |
| 10 | LB | elux vape refill blueberry | - | NDD |  |
| 11 | LB | jucce candy black jack | - | NDD |  |
| 12 | UB | unknown -in small vial no ID | Clear | MDMB-4en-PINACA |  |
| 13 | LB | pukka juice lime lemonade | - | NDD |  |
| 14 | LB | soda king lemon lime | - | NDD |  |
| 15 | LB | crystal clear blueberry sour raspberry | - | NDD |  |
| 16 | RF | - | - | MDMB-4en-PINACA |  |
| 17 | RF | OXVA gold/green with refill top | - | NDD |  |
| 18 | LB | elux vape refill strawberry ice cream | - | NDD |  |
| 19 | LB | elux vape refill kiwi passion fruit | - | NDD |  |
| 20 | LB | elux vape refill cherry cola | - | NDD |  |
| 21 | RF | unknown | - | NDD |  |
| 22 | RF | black and gold | - | MDMB-4en-PINACA |  |
| 23 | SU | pale green KLAK | - | NDD |  |
| 24 | RF | lime green and green OXVA | - | NDD |  |
| 25 | LB | elux tiger blood | - | NDD |  |
| 26 | RF | blue and black OXVA | - | NDD |  |
| 27 | LB | elflio - cherry | - | NDD |  |
| 28 | RF | silver oxva | - | MDMB-4en-PINACA |  |
| 29 | SU | - | - | NDD |  |
| 30 | RF | - | - | MDMB-4en-PINACA |  |
| 31 | RF | - | - | NDD |  |
| 32 | RF | - | - | NDD |  |
| 33 | RF | - | - | MDMB-4en-PINACA |  |
| 34 | UB | - | Pink | MDMB-4en-PINACA |  |
| 35 | RF | - | - | MDMB-4en-PINACA |  |
| 36 | RF | - | - | NDD |  |
| 37 | RF | - | - | MDMB-4en-PINACA |  |
| 38 | RF | - | - | NDD |  |
| 39 | RF | - | - | MDMB-4en-PINACA |  |
| 40 | RF | - | - | MDMB-4en-PINACA |  |
| 41 | LB | mango | - | NDD |  |
| 42 | LB | watermelon | - | NDD |  |
| 43 | LB | blueberry | - | NDD |  |
| 44 | UB | - | green | MDMB-4en-PINACA |  |
| 45 | SU | yellow with white top | - | THC |  |
| 46 | SU | crystal fire blue and yellow | - | NDD |  |
| 47 | LB | lemon and lime nearly full | - | NDD |  |
| 48 | LB | lemon and lime nearly empty | - | NDD |  |
| 49 | SU | lost mary red/yellow | - | NDD |  |
| 50 | SU | Hayati pro max | - | NDD |  |

NDD, no drug detected; THC, tetrahydrocannabinol; SU, single use; RF, refillable; UB, unlabelled bottle; LB, labelled bottle.

Table S6. Data summary for R1, R2 and R3 for SC positives.

| School | SC detected | Total submission | Total single use | Total liquid/refillable | Total positive | Single use positive | Liquid/refillable positive | [Average] (mg / mL) | Total positive rate | Single use positive rate | Liquid/refillable positive rate |
| --- | --- | --- | --- | --- | --- | --- | --- | --- | --- | --- | --- |
|  |  |  |  |  |  |  |  |  |  |  |  |
| R1S1 | Yes | 9 | 0 | 9 | 5 | 0 | 5 | 0.55 | 0.56 | 0.00 | 0.56 |
| R1S2 | Yes | 5 | 2 | 3 | 3 | 0 | 3 | 1.37 | 0.60 | 0.00 | 0.60 |
| R1S3 | No | 1 | 1 | 0 | 0 | 0 | 0 | 0.00 | 0.00 | 0.00 | 0.00 |
| R1S4 | Yes | 5 | 4 | 1 | 1 | 0 | 1 | 0.56 | 0.20 | 0.00 | 0.20 |
| R1S5 | No | 3 | 3 | 0 | 0 | 0 | 0 | 0.00 | 0.00 | 0.00 | 0.00 |
| R1S6 | Yes | 4 | 2 | 2 | 2 | 0 | 2 | 0.40 | 0.50 | 0.00 | 0.50 |
| R1S7 | Yes | 26 | 23 | 3 | 3 | 0 | 3 | 2.04 | 0.12 | 0.00 | 0.12 |
| R1S8 | Yes | 36 | 16 | 20 | 10 | 1 | 9 | 1.40 | 0.28 | 0.03 | 0.25 |
| R1S9 | Yes | 6 | 1 | 5 | 4 | 0 | 4 | 1.16 | 0.67 | 0.00 | 0.67 |
| R1S10 | No | 21 | 21 | 0 | 0 | 0 | 0 | 0.00 | 0.00 | 0.00 | 0.00 |
| R1S11 | Yes | 1 | 0 | 1 | 1 | 0 | 1 | 1.50 | 1.00 | 0.00 | 1.00 |
| R1S12 | Yes | 33 | 27 | 6 | 6 | 0 | 6 | 1.25 | 0.18 | 0.00 | 0.18 |
| R1S13 | No | 6 | 6 | 0 | 0 | 0 | 0 | 0.00 | 0.00 | 0.00 | 0.00 |
| **R1** |  | **156** | **106** | **50** | **35** | **1** | **34** | **0.00** | **0.224** | **0.01** | **0.22** |
|  |  |  |  |  |  |  |  |  |  |  |  |
| R2S1 | Yes | 31 | 24 | 7 | 12 | 6 | 6 | 0.88 | 0.39 | 0.19 | 0.19 |
| R2S2 | Yes | 32 | 20 | 12 | 3 | 0 | 3 | 0.34 | 0.09 | 0.00 | 0.09 |
| R2S3 | Yes | 99 | 71 | 28 | 13 | 0 | 13 | 0.76 | 0.13 | 0.00 | 0.13 |
| R2S4 | Yes | 3 | 0 | 3 | 2 | 0 | 2 | 0 | 0.67 | 0.00 | 0.67 |
| R2S5 | Yes | 25 | 20 | 5 | 1 | 0 | 1 | 0 | 0.04 | 0.00 | 0.04 |
| R2S6 | Yes | 6 | 3 | 3 | 3 | 0 | 3 | 1.86 | 0.50 | 0.00 | 0.50 |
| R2S7 | No | 7 | 4 | 3 | 0 | 0 | 0 | 0 | 0.00 | 0.00 | 0.00 |
| R2S8 | No | 13 | 12 | 1 | 0 | 0 | 0 | 0 | 0.00 | 0.00 | 0.00 |
| R2S9 | Yes | 54 | 42 | 12 | 6 | 1 | 5 | 0.47 | 0.11 | 0.02 | 0.09 |
| **R2** |  | **270** | **196** | **74** | **40** | **7** | **33** | **0** | **0.148** | **0.03** | **0.12** |

| School | SC detected | Total submission | Total single use | Total liquid/refillable | Total positive | Single use positive | Liquid/refillable positive | [Average] (mg / mL) | Total positive rate | Single use positive rate | Liquid/refillable positive rate |
| --- | --- | --- | --- | --- | --- | --- | --- | --- | --- | --- | --- |
| R3S1 | Yes | 15 | 7 | 8 | 1 | 0 | 1 | NT | 0.07 | 0.00 | 0.07 |
| R3S2 | Yes | 26 | 14 | 12 | 5 | 0 | 5 | NT | 0.19 | 0.00 | 0.19 |
| R3S3 | Yes | 7 | 0 | 7 | 6 | 0 | 6 | NT | 0.86 | 0.00 | 0.86 |
| R3S4 | Yes | 1 | 0 | 1 | 1 | 0 | 1 | NT | 1.00 | 0.00 | 1.00 |
| R3S5 | Yes | 35 | 16 | 19 | 1 | 0 | 1 | NT | 0.03 | 0.00 | 0.03 |
| **R3** |  | **84** | **37** | **47** | **14** | **0** | **14** | **NT** | **0.167** | **0.00** | **0.167** |
| **R1+R2+R3** | **0.75** | **510** | **339** | **171** | **89** | **8** | **81** | **1.03** | **0.1745** | **0.0157** | **0.159** |
|  |  |  |  |  |  |  |  |  |  |  |  |
|  |  |  |  |  |  |  |  |  |  |  |  |
|  |  |  |  |  |  |  |  |  |  |  |  |
|  |  |  |  |  |  |  |  |  |  |  |  |
|  |  |  |  |  |  |  |  |  |  |  |  |
|  |  |  |  |  |  |  |  |  |  |  |  |
|  |  |  |  |  |  |  |  |  |  |  |  |
|  |  |  |  |  |  |  |  |  |  |  |  |
|  |  |  |  |  |  |  |  |  |  |  |  |
|  |  |  |  |  |  |  |  |  |  |  |  |
|  |  |  |  |  |  |  |  |  |  |  |  |
|  |  |  |  |  |  |  |  |  |  |  |  |
|  |  |  |  |  |  |  |  |  |  |  |  |
|  |  |  |  |  |  |  |  |  |  |  |  |
|  |  |  |  |  |  |  |  |  |  |  |  |
|  |  |  |  |  |  |  |  |  |  |  |  |

NT, not tested

Table S7. LC-MS parent molecule and fragment ion mass to charge (mz) ratios used to confirm compounds found in the e-cigarettes/liquids.

| Compound | Parent | F1 | F2 | F3 | F4 | F5 | F6 |
| --- | --- | --- | --- | --- | --- | --- | --- |
| MDMB-4en-PINACA | 358.2125 | 213.1022 | 298.1914 | 145.0396 | 86.0964 | 171.089 | 163.0502 |
| ADB-BUTINACA | 331.2128 | 201.1022 | 286.1914 | 314.1863 | 163.0502 | 145.0396 | 219.1128 |
| 4F-MDMB-BINACA | 364.2031 | 219.0928 | 304.1819 | 145.0396 | 237.1034 | 237.1022 | 236.1193 |
| MDMB-PINACA | 360.2281 | 215.1179 | 145.0396 | 300.207 |  |  |  |
| ADB-4en-PINACA | 343.2128 | 213.1022 | 298.1914 | 326.1863 | 365.1948 |  |  |
| MDMB-INACA | 290.1499 | 145.0396 | 230.1288 | 86.0964 |  |  |  |
| Δ9-THC | 315.2131 | 193.1223 | 123.0441 | 259.1693 | 235.1693 | 233.1536 | 221.1536 |

**
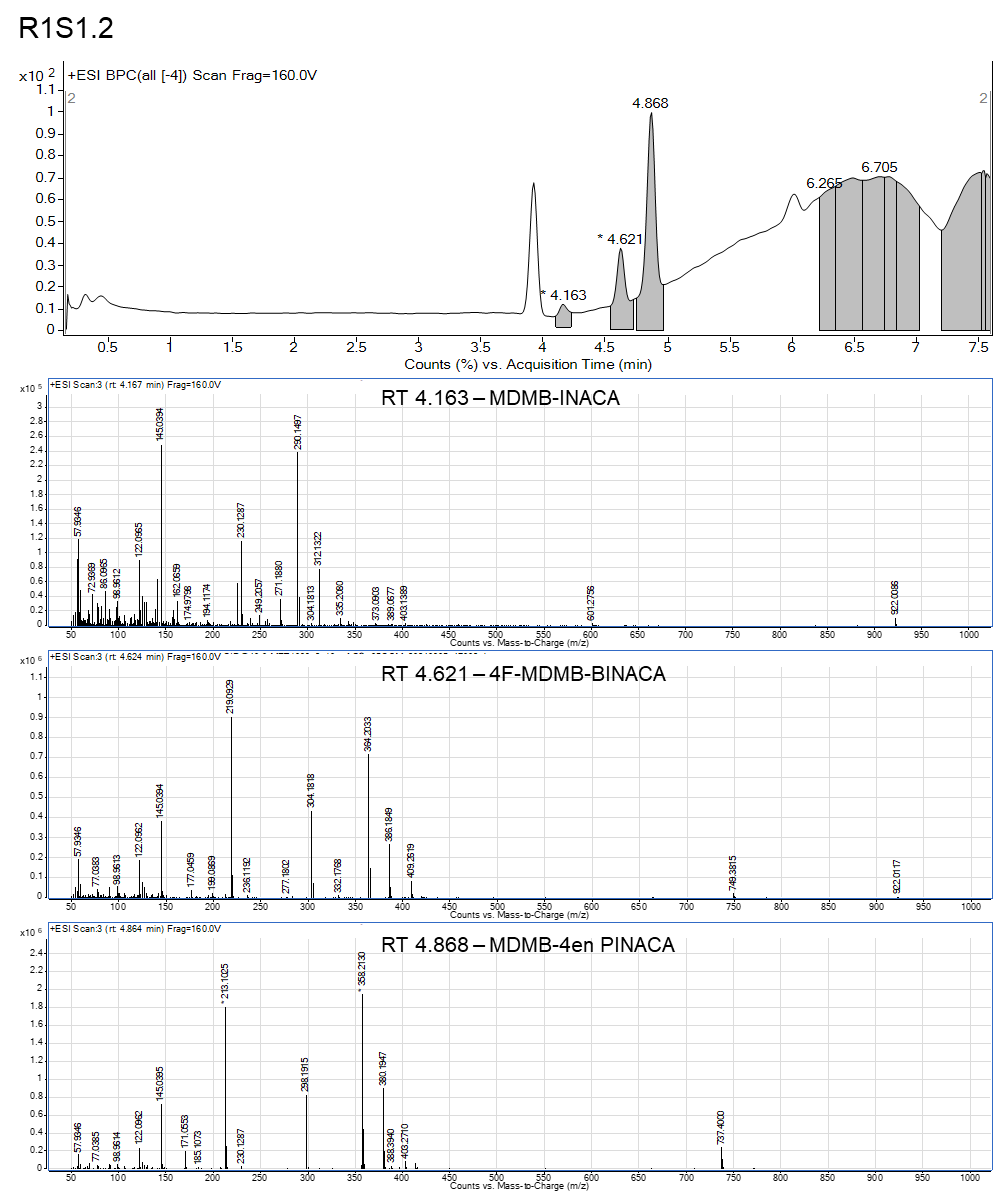
**

**Figure S1.** Example LC-MS chromatogram and spectra for sample R1S1.2 with MDMB-4en-PINACA, 4F-MDMB-BINACA and MDMB-INACA SCs identified. Molecular weights of compounds: MDMB-INACA – 289.330 g mol^-1^, 4F-MDMB-BINACA – 363.433 g mol^-1^, MDMB-4en-PINACA - 357.454 g mol^-1^.

**
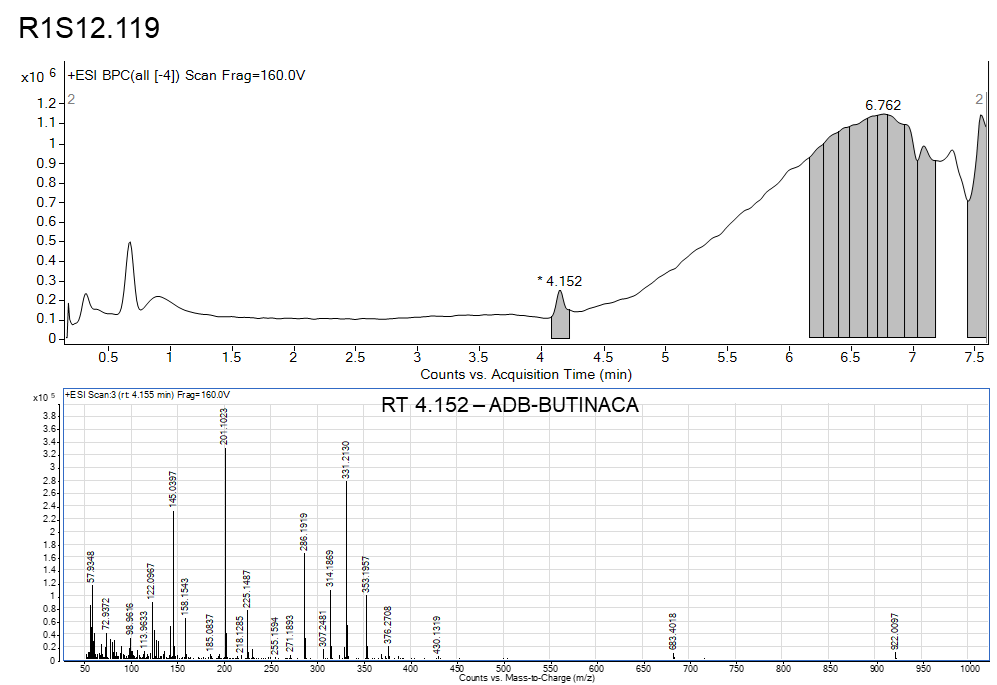
**

**Figure S2.** Example LC-MS chromatogram and spectra for sample R1S12.119 with ADB-BUTINACA identified. Molecular weight of ADB-BUTINACA is 330.432 g mol^-1^.

**
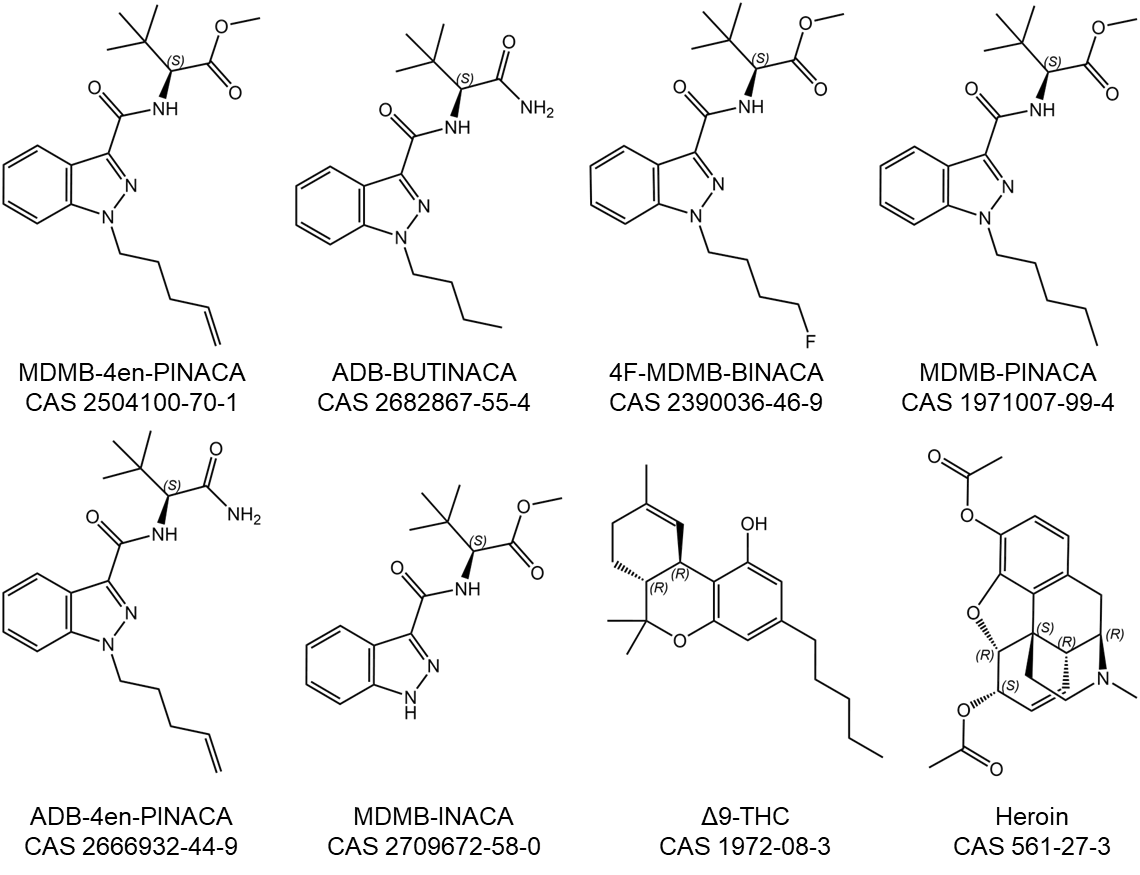
**

**Figure S3.** Structures of illicit drugs identified.

**
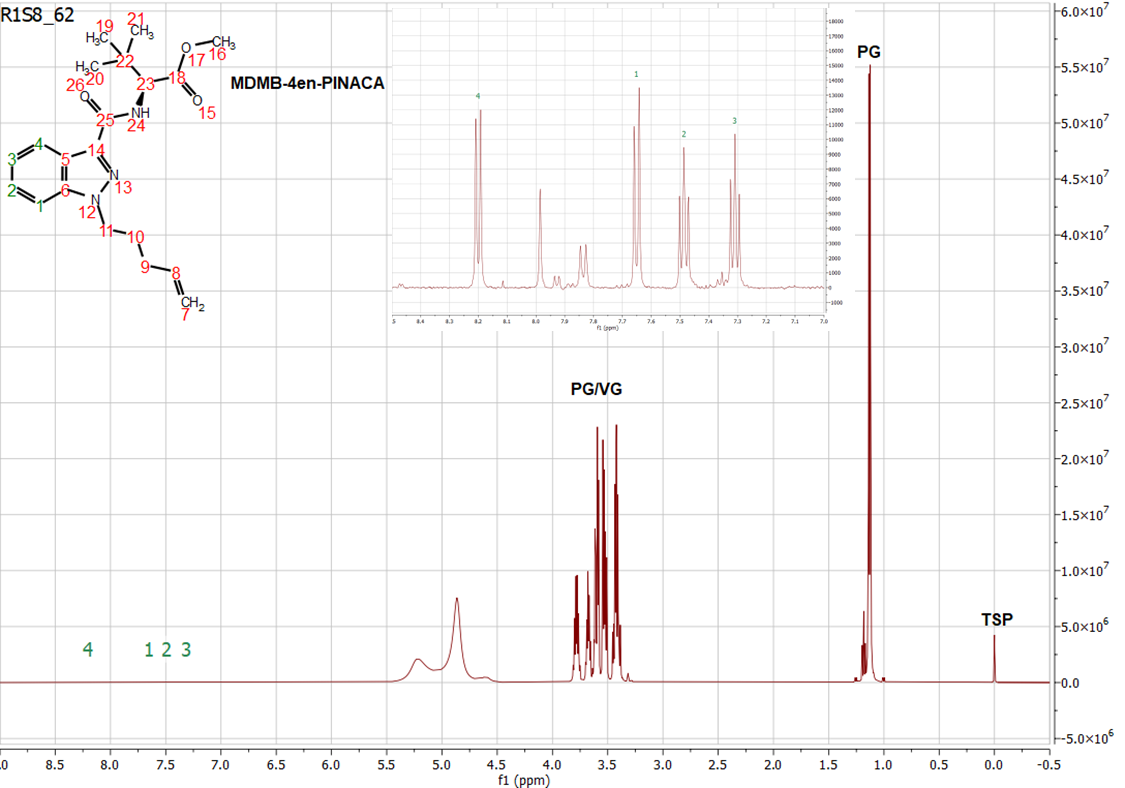
**

**Figure S4.** Example 1H NMR spectra used for qNMR on sample R1S8.62 containing MDMB-4en-PINACA. Main spectra shows the sample is dominated by propylene glycol (PG) and vegetable glycerin (VG) that form the basis of e-cigarette liquid. Insert is of the 8.5 – 7.0 ppm region which shows the aromatic indazole peaks for hydrogens 1-4 used for the quantification. The internal standard 3-(trimethylsilyl)propionic-2,2,3,3-d4 acid sodium salt (TSP) is shown at 0.0 ppm (3 mg added). The qNMR method used a 20 s delay, 128 scans and processing techniques (as described in materials and methods) such that ratio of the integration of the sample to TSP peaks can be used to calculate the concentration of the sample, 3.53 mg/mL in this case.

**
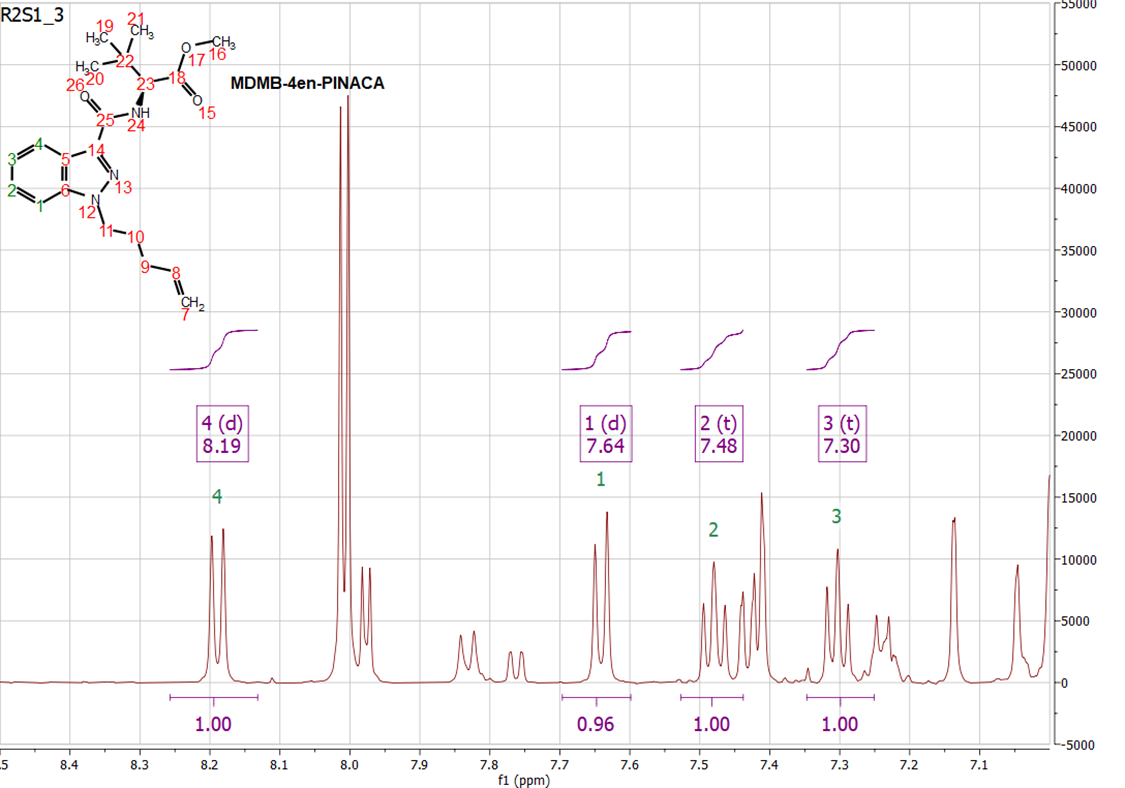
**

**Figure S5.** Example 1H NMR spectra used for qNMR on sample R2S1.3 containing MDMB-4en-PINACA. The aromatic indazole 8.5 – 7.0 ppm region is shown with the multiplets and integration of hydrogens 1-4 used for the quantification.

**
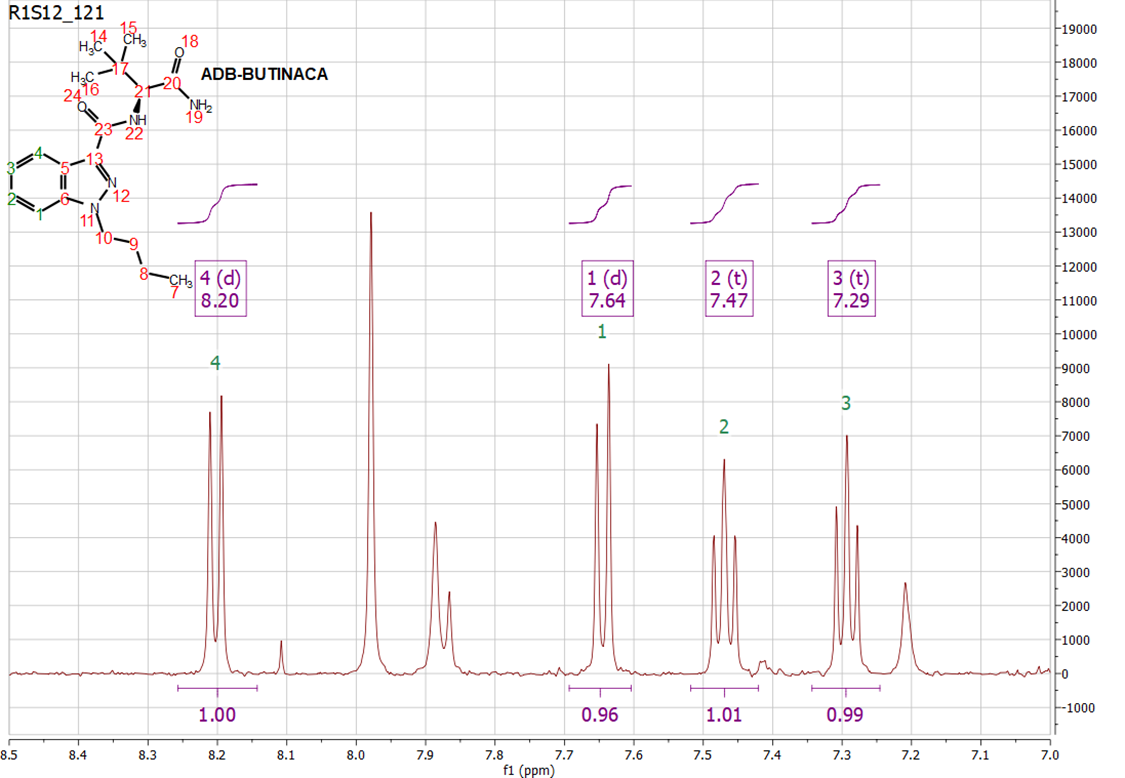
**

**Figure S6.** Example 1H NMR spectra used for qNMR on sample R1S12.121 containing ADB-BUTINACA. The aromatic indazole 8.5 – 7.0 ppm region is shown with the multiplets and integration of hydrogens 1-4 used for the quantification.

**
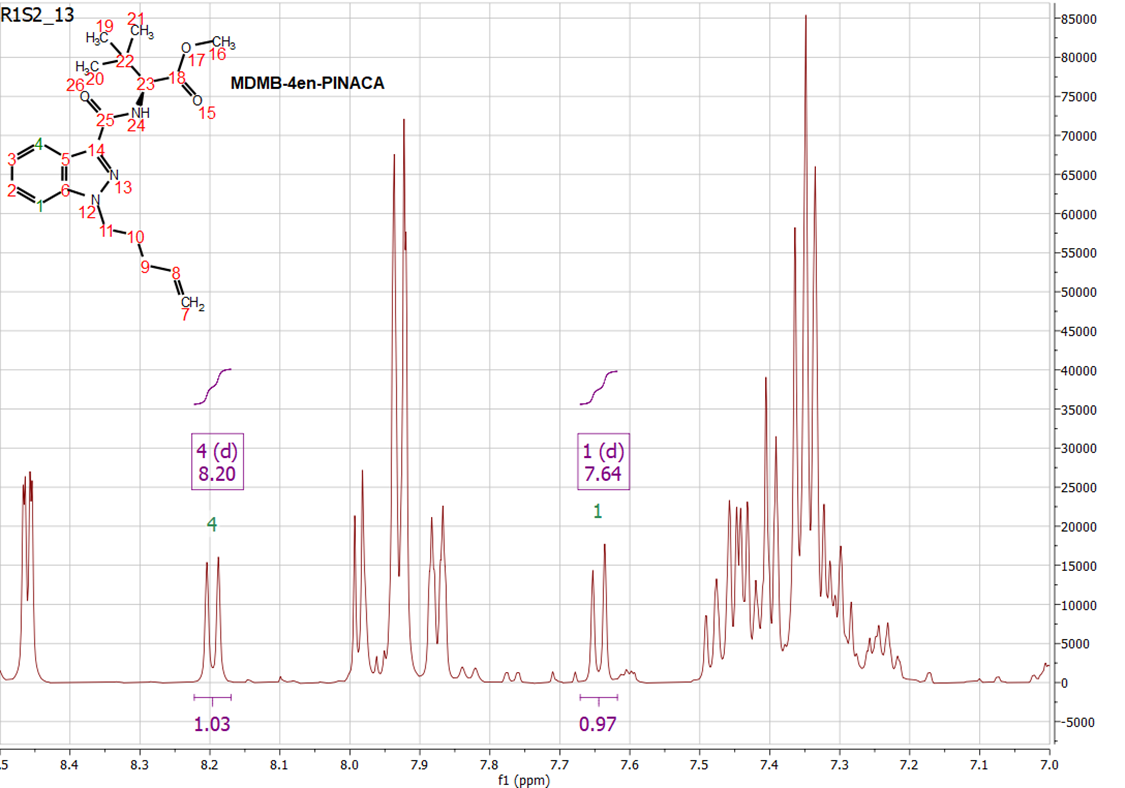
**

**Figure S7.** Example 1H NMR spectra used for qNMR on sample R1S2.13 containing MDMB-4en-PINACA. The aromatic indazole 8.5 – 7.0 ppm region is shown highlighting that only the multiplets and integration of hydrogens 1 and 4 are able to be used for quantification due to additives in the e-cigarette liquid masking the other hydrogens.

**
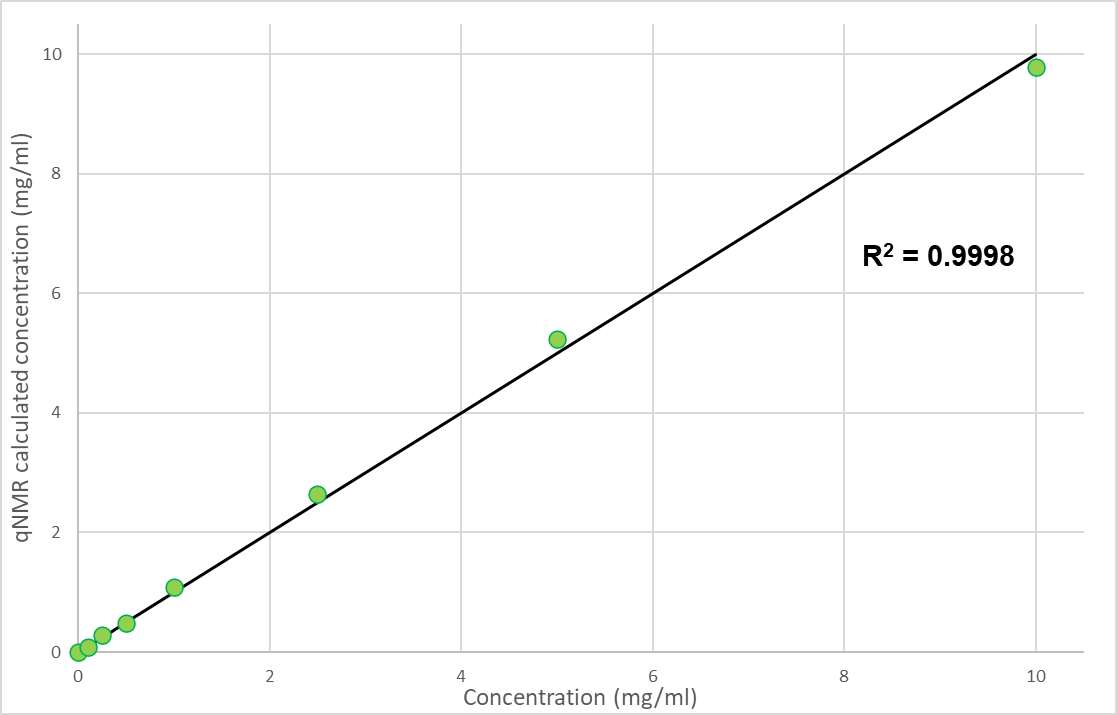
**

**Figure S8.** Plot showing the qNMR calculated values of a standard concentration range of MDMB-4en-PINACA in e-cigarette liquid. This shows good agreement with the true values (black line) highlighting the suitability of the qNMR technique for quantifying the e-cigarettes and liquids.

**
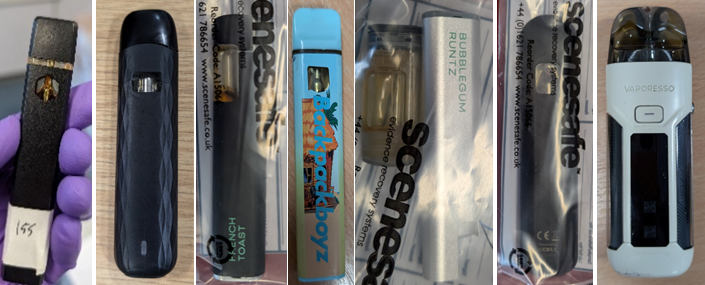
**

**Figure S9.** THC e-cigarettes from R3.

**
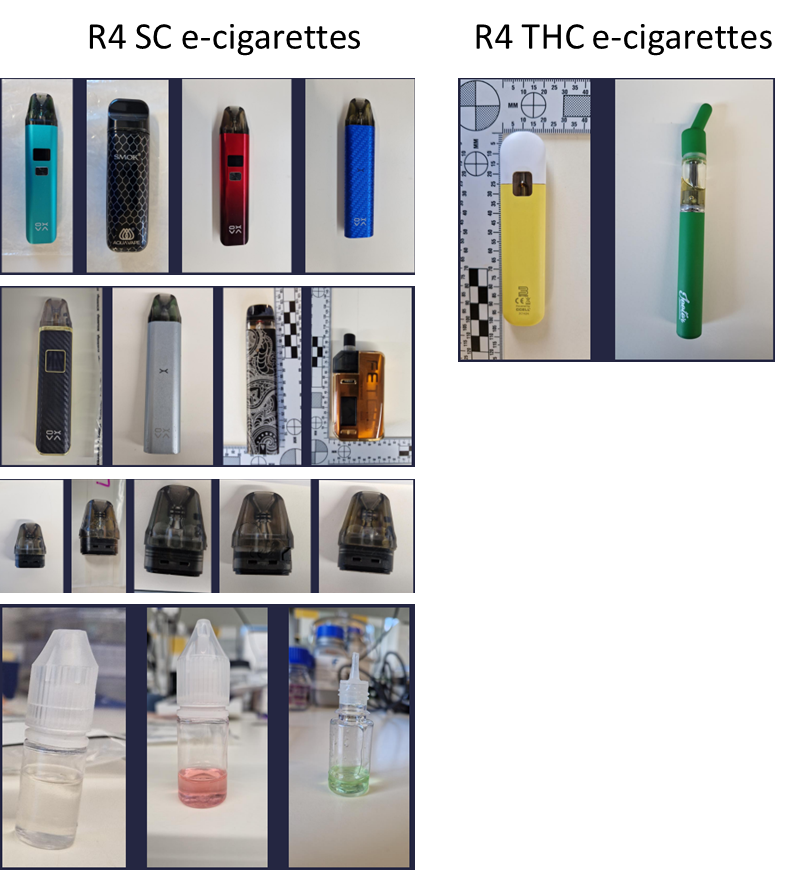
**

**Figure S10.** Positive samples (SC and THC) from R4.
